# Astrocyte-derived complement C3 facilitated microglial phagocytosis of synapses in *Staphylococcus aureus*-associated neurocognitive deficits

**DOI:** 10.1371/journal.ppat.1013126

**Published:** 2025-04-28

**Authors:** Haifang Zhang, Qiyuan Jin, Jijie Li, Jiali Wang, Mengqi Li, Qiao Yin, Qi Li, Yuwan Qi, Lingling Feng, Liang Shen, Yuan Qin, Qifei Cong

**Affiliations:** 1 Department of Clinical Laboratory, The Second Affiliated Hospital of Soochow University, Suzhou, China; 2 MOE Key Laboratory of Geriatric Diseases and Immunology, Soochow University, Suzhou, China; 3 Institute of Neuroscience and Jiangsu Key Laboratory of Neuropsychiatric Diseases, Soochow University, Suzhou, China; 4 Department of Neurology, Clinical Research Center of Neurological Disease, The Second Affiliated Hospital of Soochow University, Suzhou, China; 5 Department of Nephrology, The Second Affiliated Hospital of Soochow University, Suzhou, China; University of Illinois at Chicago College of Medicine, UNITED STATES OF AMERICA

## Abstract

The presence of pathogens is a significant challenge in causing brain infections and tissue damage. There is growing evidence that pathogen infections are commonly associated with cognitive dysfunction and mental health problems, but the underlying mechanisms are not yet fully understood. Here, we found microglia and astrocyte activation, neuronal damage, synapse loss, and cognitive impairment in a *Staphylococcus aureus* (*S. aureus*) induced mouse model. An unbiased transcription profile of isolated microglia derived from *S. aureus*-infected mice identified the involvement of microglial phagosome and regulation of neurogenesis. Our findings indicate that the complement C1q and C3 are upregulated, and astroglial release of C3 activates microglia to phagocytose synapses. Blocking the C3-C3aR axis can improve microglial phagocytosis, thus rescuing synapse loss and cognitive impairment in infected mice. These results indicate that *S. aureus* induces synapse elimination and cognitive impairment by activating microglia and astrocytes through C3-C3aR signaling. This suggests a mechanism of complement signaling bridged crosstalk between astrocyte and microglia in the *S. aureus*-associated post-infectious synapse loss and cognitive dysfunction, and provide potential therapeutic targets for managing pathogen-associated brain infections.

## Author summary

Infections caused by pathogens can lead to serious brain complications, including cognitive issues and mental health challenges. However, the exact mechanisms behind these effects remain unclear. Our study used a mouse model infected with *S. aureus* to explore how this bacterium impacts brain function. We discovered that the infection leads to the activation of two key brain cell types, microglia and astrocytes, which causes damage to neurons, loss of connections between brain cells (synapses), and cognitive decline. This study identified that the complement system, particularly proteins C1q and C3, plays a crucial role in this process. Specifically, C3 released by astrocytes activates microglia, leading them to consume and eliminate synapses. Importantly, we found that blocking the C3-C3aR pathway could prevent this synapse loss and improve cognitive function in the infected mice. These findings shed light on how brain cells communicate during infection and suggest new avenues for treating brain injury caused by pathogens.

## Introduction

Infections caused by microorganisms, including bacteria, viruses, fungi, or parasites, are leading to a global burden of morbidity and mortality. The neurological complications resulting from infections, including cognitive and mental health issues, present a significant challenge for millions of children and adults in low- and middle-income countries [1]. Growing evidence has shown that individuals are susceptible to microcephaly and severe neurological malformations following Zika virus infection for pregnant women and their fetuses [2–4]. Human immunodeficiency virus has the potential to induce acquired neurocognitive impairment, impacting between 30–50% of the 38 million individuals worldwide [5]. West Nile virus infection can result in persistent cognitive impairment and functional decline even in patients who recover from West Nile Virus (WNV) neuroinvasive disease [6,7]. Zika virus infection in the mature central nervous system causes synaptic dysfunction, microglia activation, and memory dysfunction [8]. Common bacterial infections impacting the nervous system include neonatal meningitis and sepsis. These conditions are associated with potential neurological and cognitive impairment [1,9]. The most common bacterial infections affecting the nervous system are sepsis and meningitis in neonates [1]. Neonatal meningitis and neonatal sepsis are associated with long-term neurological and cognitive impairment, primarily impairment of hearing, vision, or motor function, cerebral palsy, and epilepsy. Meanwhile, neonatal sepsis and meningitis have been linked to long-term neurological and cognitive impairment, particularly affecting hearing, vision, and motor function, and may also lead to conditions such as cerebral palsy and epilepsy. The leading causes of sepsis and meningitis in neonates are derived from prevalent bacterial infections, including *Staphylococcus aureus*, *Escherichia coli*, and *Klebsiella pneumonia* [1,10–12]. During the first month of life, it’s important to note that pneumococcal meningitis, caused by *Streptococcus pneumoniae*, represents about 9% of neonatal bacterial meningitis cases. Meanwhile, meningococcal meningitis, caused by *Neisseria meningitidis*, accounts for approximately 8% of these cases [13]. *Streptococcus agalactiae*, often known as group B *Streptococcus* (GBS), is recognized as the most prevalent bacterial cause of meningitis in the neonatal period. This highlights the critical need to understand pathogen-specific mechanisms of blood-brain barrier penetration and neuroinvasion [14]. Bacterial pathogens can infiltrate the meninges and the brain via the blood-brain barrier (BBB) or the olfactory nervous system, potentially resulting in significant pathological effects [15,16]. Despite the severe pathology that bacteria can cause to the nervous system, there is limited understanding of the response in the central nervous system to bacterial infection and the resulting impact on behavior and neurological manifestations.

*Staphylococcus aureus* (*S. aureus*, SA) is a Gram-positive opportunistic pathogen that can lead to invasive infections such as brain abscess, pneumonia, arthritis, and endocarditis [17]. *S. aureus* colonizes the nasal passages or skin of approximately 20% of healthy adults [17,18]. The emergence of methicillin-resistant *S. aureus* (MRSA) and vancomycin-intermediate *S. aureus* (VISA) has exacerbated the problem of bacterial drug resistance, which can lead to treatment failure in *S. aureus* infections [19,20]. A recent study revealed that 18% of culture-positive brain abscesses were due to *Staphylococcus* infections [21]. Surgical site infections occur in 1–3% of craniotomy surgeries, half caused by *S. aureus* [22,23].

Microglia are the resident macrophages in the central nervous system responsible for phagocytosis of cellular debris, production of inflammatory responses, and modulation of synaptic plasticity [24–27]. When neurons are stressed and express ‘find-me’ signals, activated microglia migrate to the injury site and engulf damaged cells that express ‘eat-me’ signals [28]. Phosphatidylserine and complement proteins C1Q and C3 and their multiple interacting regulators have been found to serve as the ‘eat-me’ signals [29–31]. A feature of activated microglia is the activation of complement cascade, which belongs to the innate immune system typically utilized for the phagocytosis of pathogens or cellular debris [32,33]. Moreover, the complement system is also an immune barrier against pathogen infection. In the innate immune system, the three distinct complement pathways initiated by complement C1Q, mannan-binding lectin (MBL), or C3 undergoing spontaneous hydrolysis induce the formation of the C3 convertase and converge to cleave and activate the central complement C3, leading to the binding of pathogens and cellular debris [34]. This process ultimately results in cell lysis or clearance by phagocytic cells. Complement proteins C3 and C1q are involved in microglia-mediated synaptic pruning during neuronal development [31,35] and synaptic loss in neurodegenerative processes [8,36]. Notably, the complement protein C1QA exhibited an increase and was found to be localized to microglia, infected neurons, and presynaptic terminals during WNV neuroinvasive disease. Deletion of C3 or C3a receptor (C3aR) in mice protected WNV-induced synapse loss and spatial memory impairment [37]. The production of complement C3 is elevated to eliminate the pathogen by microglial phagocytosis when neonatal meningitis-causing *Escherichia coli* invades the brain [38]. However, the underlying mechanism of complement-mediated microglia phagocytosis of synapses for cognitive deficits caused by *S. aureus* is still unclear.

In this study, we found microglia and astrocyte activation, neuronal damage, and synaptic loss in the mouse brain infected by *S. aureus*. Cognitive and memory impairment were detected in *S. aureus*-infected mice. We found that astrocytes secreted large amounts of C3 and upregulated classical complement system expression after bacterial infection. By knocking the complement C3-C3aR axis, synaptic damage and cognitive impairment in *S. aureus*-infected mice were rescued. These findings highlight the mechanism underlying cognitive impairment following *S. aureus* and provide potential therapeutic targets and strategies for treating brain infection.

## Results

### *S. aureus* induces abnormal behavior and neuronal damage

**Fig 1:**
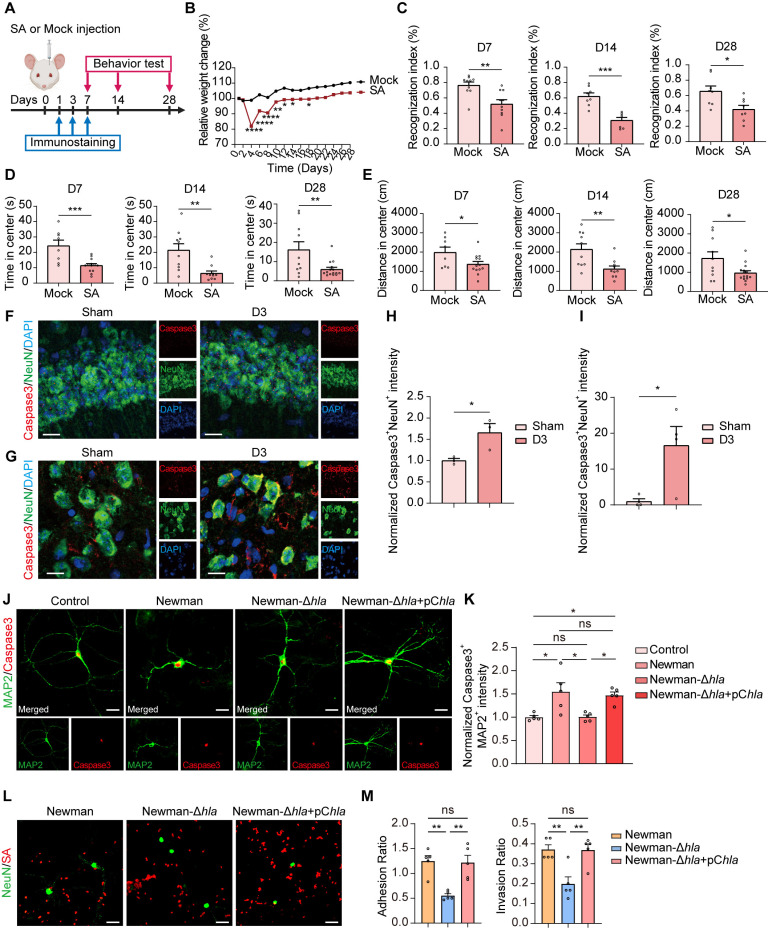
*S. aureus*–mediated cognitive impairment and damaged neurons post-infection. (A) Experimental paradigm for experiment design. Created with BioRender.com. (B) Relative change in body weight of *S. aureus*-infected mice (SA) and control (Mock) over one month. n = 9 mice (Mock) and 15 mice (SA). (C–E) Behavioral outcomes on D7, D14 and D28 in mice infected with *S. aureus* (SA) and control (Mock). Recognition index (%) in the NOR test (C). Center time (s) traveled in the open field test (D). Center distance (cm) traveled in the open field test (E). Climbing time (s) in the pole test (E). n = 9–15 mice per group. (F and G) Representative images of immunostaining for Caspase3 (red), NeuN (green), and DAPI (blue) in the hippocampus (F) and striatum (G) from C57BL/6 mice of Sham and *S. aureus* infection by 3 days (D3). Scale bar = 15 μm. (H and I) Quantification of normalized Caspase3^+^NeuN^+^ fluorescence intensity in the hippocampus (H) and striatum (I). n = 4 mice per group. (J) Representative images of immunostaining for Caspase3 (red) and MAP2 (green) in the primary hippocampal neurons treated with vehicle control, *S. aureus* wild-type strain (Newman), mutant strains lacking *hla* (Newman-Δ*hla*), and the *hla* complemented strain (Newman-Δ*hla*+pC*hla*) for two hours. Scale bar = 20 μm. (K) Quantification of normalized Caspase3^+^MAP2^+^ fluorescence intensity in primary hippocampal neuron culture. n = 5 replicates per group. (L) Representative images of immunostaining for NeuN (green) and SA (red) in the primary hippocampal neurons treated with vehicle control, *S. aureus* wild-type strain (Newman), mutant strains lacking *hla* (Newman-Δ*hla*), and the *hla* complemented strain (Newman-Δ*hla*+pC*hla*) for two hours. Scale bar = 20 μm. (M) Quantification of adhesion and invasion ratio of bacteria by primary hippocampal neurons. Adhesion ratio was calculated as [CFU of adhered bacteria]/[CFU of (non−adhered bacteria + adhered bacteria)]. Invasion ratio was calculated as [CFU of invaded bacteria]/ [CFU of (invaded bacteria + adhered bacteria)]. n = 5 replicates per group. Data are represented as mean ± SEM. Two-way ANOVA with Šídák’s multiple comparisons test for (B) and Tukey’s multiple comparisons test for (K). Unpaired Student’s t-test for (C-E, H-I), One-way ANOVA with Tukey’s multiple comparisons test for (M), *p < 0.05, **p < 0.01, ***p < 0.001, and ****p < 0.0001.

To investigate the effect of *S. aureus* infection on the central nervous system, we employed the *S. aureus* strain USA300 to generate agarose beads infused into the striatum, as previously reported [39]. Tissue samples were collected on Days 1, 3, and 7 (D1, D3, and D7) after the *S. aureus* infection (Fig 1A). Following the infusion of *S. aureus* into the striatum, motor function (striatum) and cognitive/memory processing (frontal cortex and hippocampus) behavior were tested at D7, D14, and D28 post-infection [8]. The body weights of mice were carefully monitored over a period of 28 days. During the first two weeks, mice infected with USA300 (1 × 10^5^ CFU) demonstrated a significant decrease in body weight compared to the Mock group (Fig 1B). Memory performance was examined by the novel object recognition (NOR) memory task, as demonstrated by a more extended exploration of the novel object over the familiar one. *S. aureus*-infected mice failed the NOR task (Fig 1C), indicating *S. aureus* infection impacted memory impairment. To explore the possibility of *S. aureus* infection on sickness behavior and its consequent effect on memory function, a batch of tests was conducted on mice to evaluate their locomotor/exploratory activities. As expected, both experimental groups showed the expected habituation behavior and similar distances traveled in the open-field test (OFT), but *S. aureus*-infected mice showed a shorter duration time in the center of the open-field arena compared to mock-infused mice (Fig 1D and 1E). These results indicate that *S. aureus* infection caused a sickness behavior and memory impairment in mice.

It has been demonstrated that *S. aureus* can induce apoptosis in various cell types [40]. To investigate whether the abnormal behaviors result from *S. aureus*-induced neuron death in the hippocampus (cognitive/memory impairment) and striatum (motor function) regions, we immunostained the neuronal marker NeuN along with the apoptosis marker Caspase-3 in the hippocampus and striatum three days post-infection (Fig 1F and 1G). The results indicate a significant increase in Caspase-3 positive neurons, identified by the colocalization of NeuN and Caspase-3, by day 3 in the *S. aureus* infected mice (Fig 1H and 1I), suggesting that neurons are susceptible to *S. aureus* infection. Neuronal damage in brains infected by bacteria can result from a combination of factors, including the systemic inflammatory response, activation of resident microglia, and direct toxicity from bacterial components [41]. α-toxin, the primary hemolysin of *S. aureus*, is previously known to bind to specific cell surface receptors and trigger cell death through the classical apoptotic pathway at low doses, but via a necrotic pathway at high doses [40]. Here, to investigate the direct neuronal toxicity from bacteria, we assayed the effect of α-toxin from *S. aureus* on neuronal death using the primary hippocampal neurons isolated from mouse pups as previously described [42]. Primary hippocampal neurons were treated with *S. aureus* strain (Newman), mutant strains lacking α-toxin (Newman-Δ*hla*), and the α-toxin complemented strain (Newman-Δ*hla*+pC*hla*) for two hours. Cell death was evaluated by staining for Caspase-3 and MAP2 (Fig 1J). We observed that the Newman strain led to a rapid increase in neuron cell death when compared to the control group. When neurons were infected with the mutant strain Newman-Δ*hla*, there was a significant reduction in cell death compared to the Newman strain. This promising protective effect, however, was diminished in the presence of the complemented strain Newman-Δ*hla*+pC*hla* (Fig 1K). These findings suggest that the death of neuronal cells can be caused by direct bacterial interactions through α-toxin release.

To study the effect of α-toxin on the adhesion and internalization of *S. aureus* by neurons, we further infected primary hippocampal neurons with three different strains: the Newman strain, the mutant strain (Newman-Δ*hla*), and the complemented strain (Newman-Δ*hla*+pC*hla*). Afterward, we evaluated both the adhesion and internalization rates of the bacteria by staining for *S. aureus* and NeuN (Fig 1L). We found that the adhesion of the Newman strain to primary hippocampal neurons was significantly lower in the Newman-Δ*hla* mutant compared to Newman strain, suggesting that the hemolysin produced by the Newman strain may play a role in bacterial adherence. Notably, this reduction in adhesion was reversed in the complemented strain Newman-Δ*hla*+pC*hla* (Fig 1M). Furthermore, internalization assays demonstrated that the Newman-Δ*hla* mutant had a significantly diminished capability to invade primary hippocampal neurons compared to the Newman strain. Interestingly, the complemented strain Newman-Δ*hla*+pC*hla* did not exhibit this reduced internalization (Fig 1M), reinforcing that the presence of hemolysin can enhance the invasive properties of *S. aureus*. These findings indicate that *S. aureus* induces motor dysfunction and memory impairment, paralleled by neuronal damage.

### *S. aureus* infection induces glial cell activation and phagocytosis of bacteria

**Fig 2:**
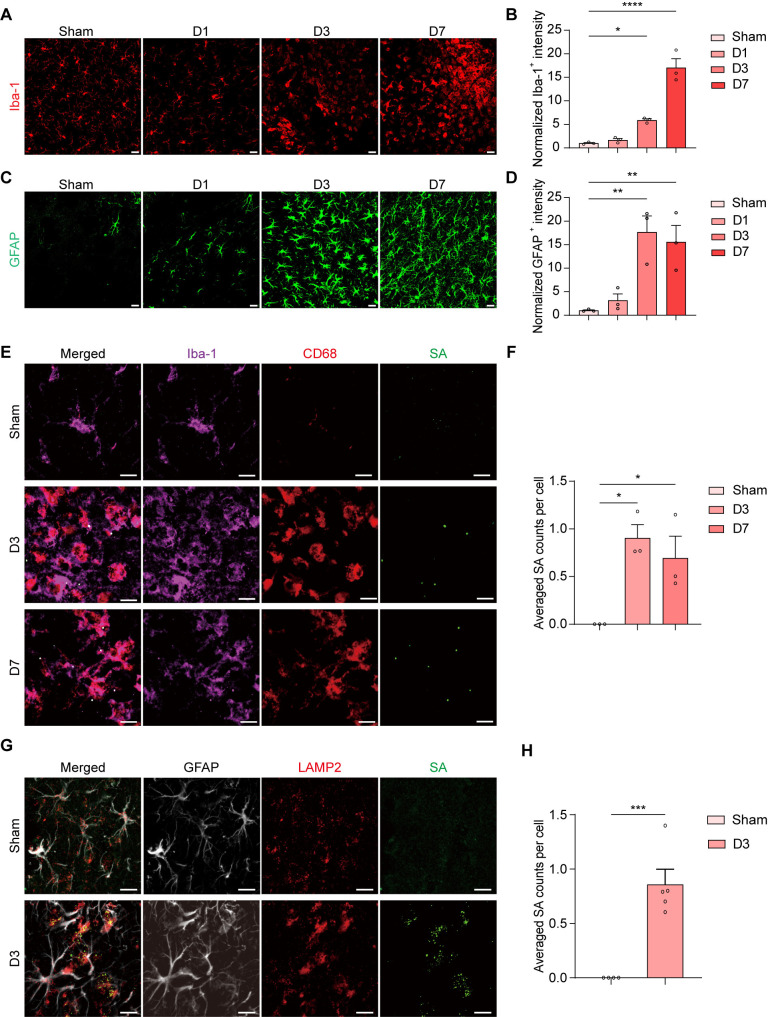
*S. aureus*–mediated cell activation and phagocytosis of bacteria by microglia and astrocytes. (A) Representative images of immunostaining for Iba-1 in the striatum from C57BL/6 mice injected with PBS (Sham) and C57BL/6 mice infected with *S. aureus* on day 1 (D1), day 3 (D3), and day 7 (D7). Scale bar = 20 μm. (B) Quantification of Iba-1^+^ fluorescence intensity. n = 3 mice per group. (C) Representative images of immunostaining for GFAP in the striatum from C57BL/6 mice injected with PBS (Sham) and C57BL/6 mice infected with *S. aureus* on day 1 (D1), day 3 (D3), and day 7 (D7). Scale bar = 20 μm. (D) Quantification of GFAP^+^ fluorescence intensity. n = 3 mice per group. (E) Representative images of immunostaining for Iba-1 (magenta), CD68 (red), and SA (green) in the striatum of C57BL/6 mice following Sham and *S. aureus* infection at 3 days (D3) and 7 days (D7). Scale bar = 15 μm. (F) Quantification of SA counts in the lysosomes (CD68) of microglia (Iba-1). n = 3 mice per group. (G) Representative images of immunostaining for GFAP (gray), LAMP2 (red), and SA (green) in the striatum of C57BL/6 mice following Sham and *S. aureus* infection at 3 days (D3). Scale bar = 15 μm. (H) Quantification of SA counts in the lysosomes (LAMP2) of astrocytes (GFAP). n = 4 - 5 mice per group. Data are represented as mean ± SEM. One-way ANOVA with Dunnett’s multiple comparisons test for (B), (D), and (F). Unpaired Student’s t-test for (H). *p < 0.05, **p < 0.01, ***p < 0.001, and ****p < 0.0001.

Next, we sought to examine whether the infection triggered gliosis. Mouse brain sections were collected at D1, D3, and D7 after *S. aureus* infection and immunostained for ionized calcium binding adaptor molecule 1 (Iba-1, a macrophage/microglial marker) and glial fibrillary acidic protein (GFAP, an astrocyte marker). No significant differences in Iba-1 and GFAP immunoreactivity were detected at day 1 in the striatum of *S. aureus*-infected mice compared to controls. In contrast, intense Iba-1 and GFAP immunoreactivity were notably increased in the brains starting from day 3 and persisted at day 7 (Fig 2A–D). To investigate if *S. aureus* infection resulted in microglial activation in specific regions, we examined microglial activation by assessing the colocalization of Iba-1 and CD68 in the contralateral striatum three days after *S. aureus* infection (S1A Fig). Our analysis indicated that microglia were activated outside the infected regions on day 3 compared to the Sham group (S1B Fig).

Microglia are primarily recognized as the primary phagocytes in the CNS. To assess the impact of activated microglia on phagocytic activity, we examined the phagocytosis of bacteria on days 3 and 7 following the *S. aureus* infection. We performed immunostaining using Iba-1, CD68 (a lysosome marker), and *S. aureus* (SA) (Fig 2E). The number of SA signals significantly increased in the microglial lysosomes from the ipsilateral striatum on day 3 and remained elevated on day 7 compared to the Sham group (Fig 2F). Additionally, astrocytes, which are non-professional phagocytes, may also play a significant role in this process [43]. We co-localized the astrocytic marker GFAP with the lysosomal marker LAMP2 and *S. aureus* (SA) (Fig 2G). Our findings showed a significant increase in LAMP2-positive phagosomes in the striatum of mice infected with *S. aureus* on day 3, compared to the Sham group. Furthermore, the presence of *S. aureus* within the astrocytic LAMP2-positive phagosomes was notably higher in the *S. aureus*-infected mice than in the Sham group (Fig 2H). Overall, these data suggest that *S. aureus* infection leads to the activation of microglia and astrocytes, resulting in increased phagocytic activity against the bacteria.

### *S. aureus* infection causes synapse loss and microglial synapse engulfment

**Fig 3:**
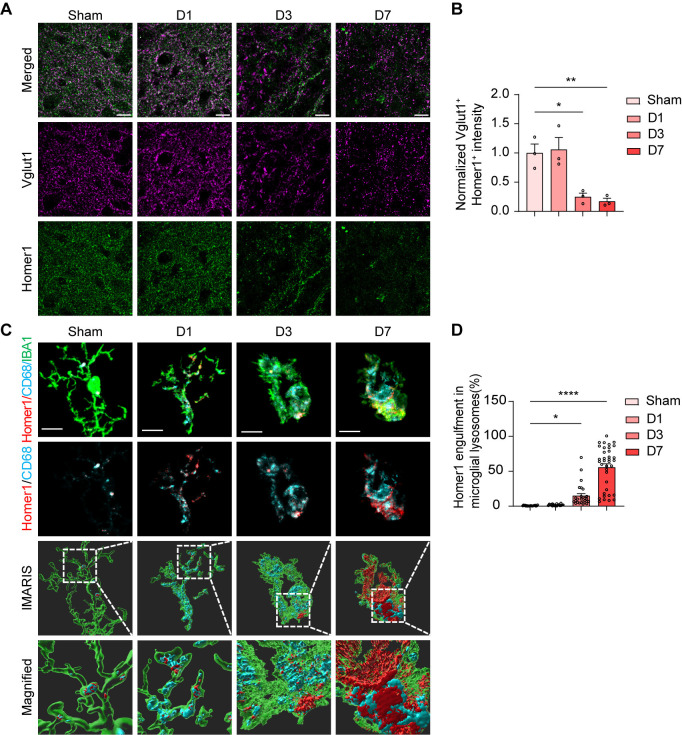
*S. aureus* caused synapse damage and increased microglial phagocytosis of synapses. (A) Representative images of excitatory presynaptic marker Vglut1 (magenta) and postsynaptic marker Homer1 (green) in the striatum from C57BL/6 mice injected with PBS (Sham) and C57BL/6 mice infected with *S. aureus* on day 1 (D1), day 3 (D3), and day 7 (D7). Scale bar = 20 μm. (B) Quantification of colocalized excitatory presynaptic marker Vglut1 (magenta) and postsynaptic marker Homer1 (green). n = 3 mice per group. (C) Representative images of microglia (green), CD68^+^ lysosomes (cyan), and postsynaptic marker Homer1 (red) in the striatum from C57BL/6 mice injected with PBS (Sham) and C57BL/6 mice infected with *S. aureus* on day 1 (D1), day 3 (D3), and day 7 (D7). Scale bar = 10 μm. (D) Quantitation of Homer1 engulfment within microglial lysosomes. n = 19 cells in Sham group of three mice, n = 18 cells in D1 group of three mice, n = 27 cells in D3 group of three mice, n = 36 cells in D7 group of three mice. Data are represented as mean ± SEM. One-way ANOVA with Dunnett’s multiple comparisons test for (B) and (D), *p < 0.05, **p < 0.01, and ****p < 0.0001.

The interaction between neurons and bacteria, along with neuroinflammatory responses in the brain, ultimately results in neuronal injury and changes to synaptic plasticity. Synaptic structural and functional plasticity, the activity-dependent change in neuronal connection strength, is widely acknowledged to play a significant role in a range of physiological and pathological processes in the adult brain, including learning, memory storage, and age-related memory decline [44–46]. Brain infections by pathogens such as the Zika virus can occur with synapse loss [8]. We thus investigated whether *S. aureus* infection would induce synapse loss. We costained with Vglut1 (a pre-synaptic marker) and Homer1 (a post-synaptic marker) at D1, D3, and D7 after *S. aureus* infection. The co-localization of Vglut1 and Homer1 immunoreactive density showed no difference from the control on D1. However, the colocalization of Vglut1 and Homer1 was dramatically decreased on D3 and D7 (Fig 3A and 3B), indicating that *S. aureus* infection resulted in synapse loss.

To further investigate whether the synapse loss induced by *S. aureus* infection in adult mice was associated with increased engulfment of synaptic components by microglia, post-synaptic marker Homer1, lysosomal marker CD68, and the microglial marker Iba-1 co-staining of brain sections was conducted on D1, D3, and D7 post-infection. A high-resolution 3D structure reconstructed by IMARIS surface rendering was utilized to analyze the relative volume of engulfed synaptic materials within the microglial lysosome. We quantified the internalization of postsynaptic membrane protein Homer1 within the lysosomes of microglia in the striatum. Statistical analysis showed no difference in the engulfed Homer1-synaptic material within microglial lysosomes on D1 but a significant increase at 3 and 7 days after *S. aureus* infection (Fig 3C and 3D). Magnified views of the microglia and phagocytosed synaptic structure revealed morphological changes and active states of microglia along with the process of infection. These results indicate that microglia extensively engulf synapses as time progresses during brain infection of *S. aureus*.

### Acute transcriptional changes of microglia following *S. aureus* infections

To understand how *S. aureus* infection affects the microglial transcriptome over time, we conducted RNA sequencing on isolated CD11b^+^ cells at D1 and D7 post-infection through magnetic-activated cell sorting (MACS) analysis. After an acute *S. aureus* infection, an in-depth analysis of the transcriptome uncovered a total of 2,770 differentially expressed genes (DEGs). Out of these, 1,900 genes showed up-regulation, while 870 genes displayed down-regulation at D1 post-infection. Furthermore, a total of 6,894 DEGs were identified, with 3,066 up-regulated and 3,828 down-regulated genes at D7 post-infection (S1 Table).

**Fig 4:**
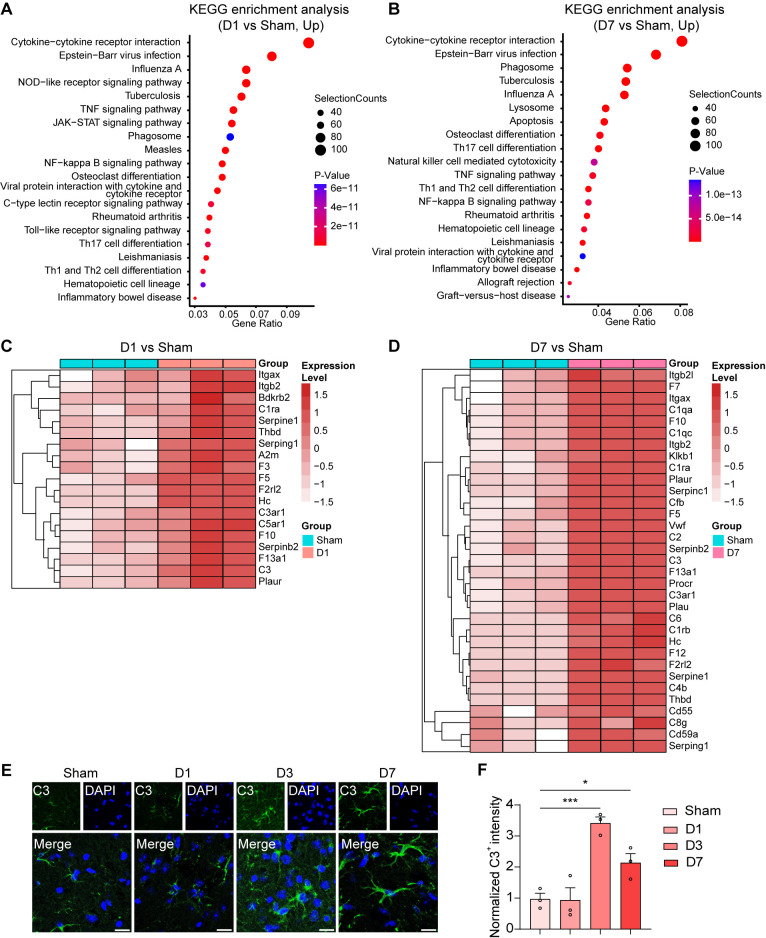
RNA-seq revealed phagocytosis and complement alteration in microglia after *S. aureus* infection. (A and B) KEGG enrichment analysis of upregulated gene expression on D1 (A) and D7 (B) after *S. aureus* infection compared to that of the Sham group. **(C and D)** Heatmap of complement and coagulation cascade pathway-associated differentially expressed genes in D1 (C) and D7 (D) after *S. aureus* infection compared to that of the Sham group. **(E)** Representative immunostaining images of C3 in the striatum from C57BL/6 mice injected with PBS (Sham) and C57BL/6 mice infected with *S. aureus* on day 1 (D1), day 3 (D3), and day 7 (D7). Scale bar = 15 μm. (F) Quantification of C3^+^ fluorescence intensity. n = 3 mice per group. Data are represented as mean ± SEM. One-way ANOVA with Dunnett’s multiple comparisons test for (F), *p < 0.05, and ***p < 0.001. See also S2 Fig and S1 Table.

Subsequent Gene Ontology (GO) analysis highlighted that the upregulated DEGs on D1 and D7 post-infection were significantly enriched in various biological processes related to immune response, including the immune system process, immune response, and regulation of immune system process (S2A and S2B Fig). Additionally, our analysis of the Kyoto Encyclopedia of Genes and Genomes (KEGG) pathways identified that the upregulated DEGs on D1 and D7 post-infection both involved in pathways including cytokine-cytokine receptor interaction, viral protein interaction with cytokine and cytokine receptor, TNF signaling pathway, NF-κB signaling pathway, Th17 cell differentiation, Th1 and Th2 cell differentiation, and phagosome (Fig 4A and 4B). Furthermore, the JAK-STAT signaling pathway, C-type lectin receptor signaling pathway, and Toll-like receptor signaling pathway were enriched at D1 post-infection. These results underscore the significant impact of acute *S. aureus* infection on the upregulated gene expression profile related to immune response at the early stage and persisting on D7, indicating enduring microglial activation and associated inflammation, characterized by an amoeboid microglia morphology in our staining. Together, our comprehensive analysis suggests the presence of sustained neuroinflammation and microglial activation in both the acute phase and later stages of *S. aureus* infection.

### Classical complement activation in microglia and astrocytes after *S. aureus* infection

The complement system is known to trigger antibacterial effects and immunity against intracellular pathogens [47,48]. Complement C1q serves as the essential protein to initiate the classical complement cascade. Upon activation, the C3 convertase further facilitates the amplification of the complement cascade by cleaving C3, resulting in various C3 fragments. These fragments play a pivotal role in promoting the activation of target cells to engage in the phagocytosis of pathogens or cell debris by interacting with specific receptors. Microglial phagocytosis is responsible for forgetting remote memories in a complement- and activity-dependent manner [49]. Given the neurocognitive deficits after *S. aureus* infection, we focused on the phagosome related to *S. aureus* infection. Our examination of the DEG enriched in phagosome allowed us to identify specific genes associated with the complement system, such as the complement *C3* gene. Additionally, we observed enrichment of the complement and coagulation cascade on days 1 and 7 post-infection (S1 Table). Furthermore, the heatmap of the DEG list highlighted the significant upregulation of multiple genes related to the complement system on D1 and D7 post-infection, including complement *C3*, *C3ar1*, *Itgax*, *Itgb2*, and *C1ra* (Fig 4C and 4D). We next assessed the expression of the classical complement system in the stratum post-infection. Western blot analysis showed a significant increase in complement C1q and C3 over time during post-infection (S2C and S2D Fig). Additionally, the expression of C3a receptor C3aR increased over time (S2C and S2D Fig), suggesting the potential involvement of the C3-C3aR pathway in this process. Moreover, the immunostaining also revealed an upregulation of complement C3 and C1q levels in the striatum post-infection by *S. aureus* (Figs 4E and 4F, and S2E and S2F), corroborating the complement activation in both the acute phase and later stages of *S. aureus* infection.

**Fig 5:**
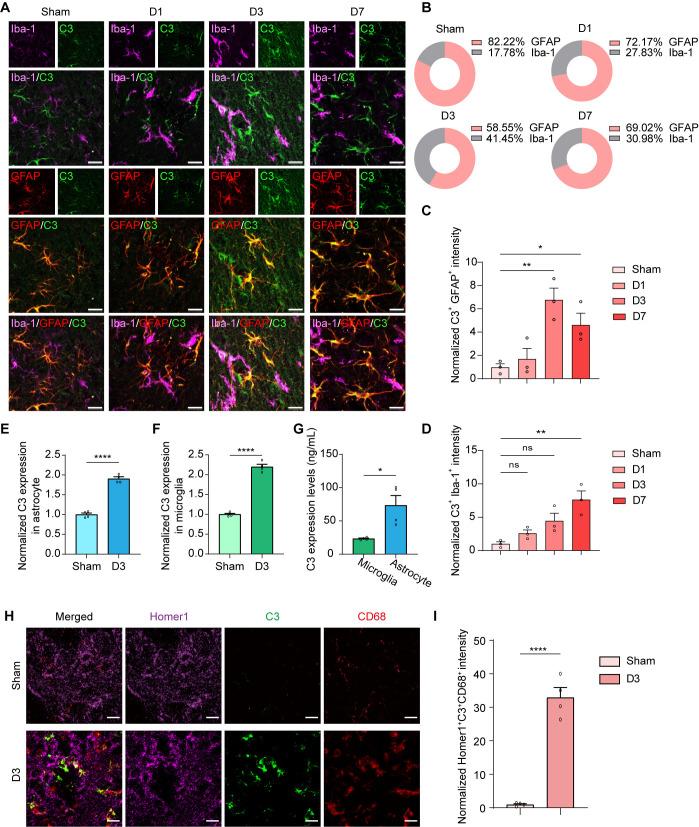
*S. aureus* infection resulted in increased C3 in astrocytes and C1q in microglia. (A) Representative images showed the expression of C3 (green) in microglia (magenta) and astrocytes (red) in the striatum from C57BL/6 mice injected with PBS (Sham) and C57BL/6 mice infected with *S. aureus* on day 1 (D1), day 3 (D3), and day 7 (D7). (B) Ratio of C3^+^Iba-1^+^ and C3^+^GFAP^+^ signals in labeled microglia and astrocyte glial cells. (C) Quantification of C3^+^GFAP^+^ signal intensity. n = 3 mice per group. (D) Quantification of C3^+^Iba-1^+^ signal intensity. n = 3 mice per group. (E and F) Quantification of C3 expression in isolated astrocytes (E) and microglia (F) from the striatum of C57BL/6 mice injected with PBS (Sham) and those infected with S. aureus on day 3 (D3) was performed using ELISA. n = 4 mice per group. (G) C3 expression levels were measured in isolated microglia and astrocytes from the striatum of C57BL/6 mice that were injected with *S. aureus* on day 3 (D3) using ELISA. n = 4 mice per group. (H) Representative images of Homer1 (magenta), C3 (green), and CD68 (red) in the striatum of C57BL/6 mice injected with PBS (Sham) and C57BL/6 mice infected with *S. aureus* on day 3 (D3). Scale bar = 15 μm. (I) Quantification of Homer^+^C3^+^CD68^+^ signals intensity. n = 4 mice per group. Data are represented as mean ± SEM. One-way ANOVA with Dunnett’s multiple comparisons test for (C) and (D). Unpaired Student’s t-test for (E, F, and I), and paired Student’s t-test for (G). *p < 0.05, **p < 0.01, ***p < 0.001 and ****p < 0.0001.

In previous studies, it has been demonstrated that a stressed, epileptic, or Alzheimer’s disease-affected brain may lead to heightened astroglial secretion of complement C3 [50–55]. In order to ascertain the origin of the escalated complement C3 levels, we conducted staining of complement C3 with astroglia cell marker (GFAP) or microglia cell marker (Iba-1) at different time points following infection. Firstly, it was observed that complement C3 predominantly originated from astrocytes in the sham group, with over 82% of C3^+^GFAP^+^ signals being in labeled microglia and astrocyte glial cells (Fig 5A and 5B). The total C3^+^GFAP^+^ and C3^+^Iba-1^+^ signals also increased significantly over time during *S. aureus* infection (Fig 5C and 5D), consistent with the changes in complement C3 levels in the astrocytic cell line C6-infected by *S. aureus* (S3A and S3B Fig). To clearly identify the primary source of complement C3, we conducted an enzyme-linked immunosorbent assay (ELISA) using MACS-isolated microglia and astrocytes from the striatum of mice infected with *S. aureus* on day 3 post-infection. Compared to the Sham group, both microglia and astrocytes exhibited an increased expression and release of complement C3 following *S. aureus* infection (Fig 5E and 5F). Notably, astrocytes showed a significantly higher level of complement C3 expression and release than microglia in the striatum affected by *S. aureus* infection (Fig 5G). These findings highlight the dominant role of astrocytes in producing complement C3 during brain injury associated with *S. aureus* infection.

Furthermore, there was an observation of increased C1q^+^ Iba-1^+^ signals in the later stage of *S. aureus* infection (S3C and S3D Fig). To better understand whether astrocyte-derived complement C3 enhances microglial synapse engulfment, we conducted immunostaining for the postsynaptic marker Homer1, the lysosome marker CD68, and complement C3 (Fig 5H). Our results showed that the presence of complement C3 colocalized with synapses was significantly increased in the lysosomes of microglia following *S. aureus* infection on day 3, compared to the Sham group (Fig 5I). This suggests a potential relationship between the enhanced complement C3 expression and secretion from astrocytes and the internalization of C3-tagged synaptic materials in microglial phagosomal compartments following the *S. aureus* infection.

### Genetic deletion of complement C3 and C3aR attenuates complement activation and microglial phagocytosis of synapses in *S. aureus*-infected mice

**Fig 6:**
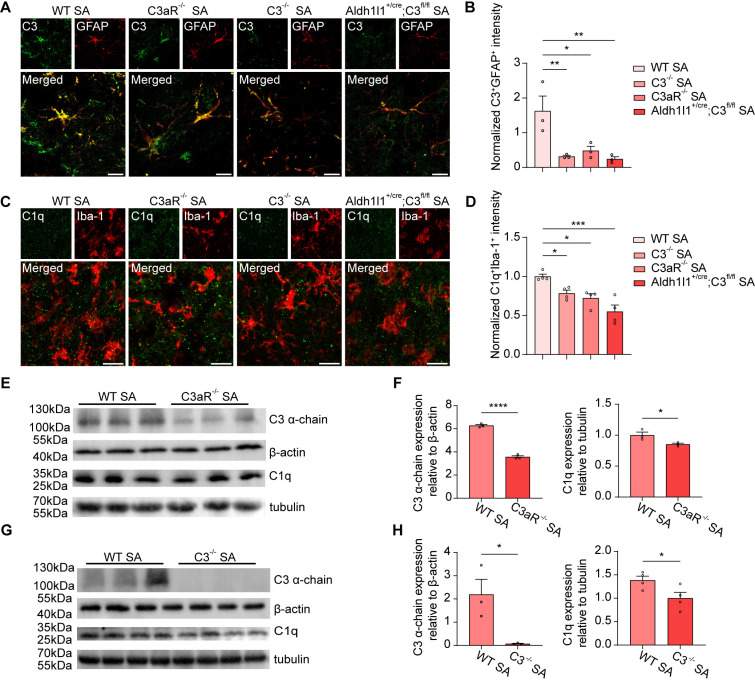
Deletion of C3 and C3aR reduced *S. aureus*-induced classical complement activation in microglia. **(A)** Representative images of C3 (green) and astrocyte (red) in the striatum of WT, C3aR^-/-^, C3^-/-^, and astrocyte-specific knockout Aldh1l1^+/cre^; C3^fl/fl^ mice after 7-day infection by *S. aureus*. **(B)** Quantification of C3^+^GFAP^+^ signals intensity. n = 3 mice per group. **(C)** Representative images of C1q (green) and microglia (red) in the striatum of WT, C3aR^-/-^, C3^-/-^, and astrocyte-specific knockout Aldh1l1^+/cre^; C3^fl/fl^ mice after 7-day infection by *S. aureus*. **(D)** Quantification of C1q ^+^Iba-1^+^ signals intensity. n = 3 mice per group. **(E and G)** Immunoblotting analysis of the complement proteins C3 α-chain and C1q in the striatum of C3aR^-/-^ (E) and C3^-/-^ (G) compared to that of WT after 7-day infection by *S. aureus*. **(F and H)** Quantification of complement proteins C3 α-chain and C1q in the striatum of C3aR^-/-^ (F) and C3^-/-^ (H) compared to that of WT after 7-day infection by *S. aureus*. n = 3 - 4 mice per group. Data are represented as mean ± SEM. One-way ANOVA with Dunnett’s multiple comparisons test for (B) and **(D)**. Unpaired Student’s t-test for (F) and **(H)**, *p < 0.05, **p < 0.01, and ****p < 0.0001.

Complement C3 is highly expressed in reactive astrocytes, where it is associated with the neurotoxic subtype implicated in AD and other neurological disorders [33,54,56]. In order to investigate the role of the complement C3-C3aR pathway in the *S. aureus*-induced infection, we employed the complete deletion of complement C3 and C3aR as well as constructed conditional astrocyte-specific C3 knockout mice (Aldh1l1^Cre-ERT2^; C3^flox/flox^). In our study, we observed a significant decrease in the intensity of C3 in GFAP-positive astrocytes in *S. aureus*-infected mice, which was almost eliminated by the deletion of C3/C3aR or by the conditional deletion of C3 in astrocytes (Fig 6A and 6B). Moreover, the intensity of C1q in Iba-1-positive microglia in *S. aureus*-infected mice was significantly reduced by C3aR or C3 deletion, which was corroborated by the reduction of C1q protein levels (Fig 6C and 6D). Furthermore, we noted reduced levels of C3 and C1q protein in the brains of genetic mice lacking C3 or C3aR (Fig 6E–H), supporting our initial observations. Overall, these results suggest that C3aR or C3 ablation can ameliorate complement activation.

**Fig 7:**
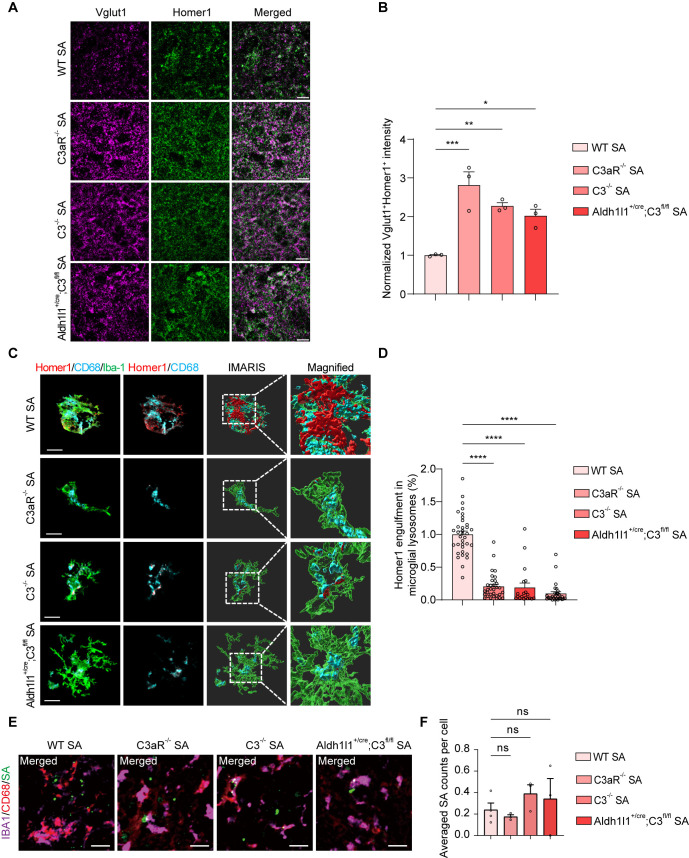
Deletion of C3 and C3aR reversed *S. aureus*-induced decrease in synapse density and increase in microglial phagocytosis of synapses. **(A)** Representative images of excitatory presynaptic marker Vglut1 (magenta) and postsynaptic marker Homer1 (green) in the striatum of WT, C3aR^-/-^, C3^-/-^, and astrocyte-specific knockout Aldh1l1^+/cre^; C3^fl/fl^ mice after 7-day infection by *S. aureus*. Scale bar = 15 μm. **(B)** Quantification of colocalized excitatory presynaptic marker Vglut1 (magenta) and postsynaptic marker Homer1 (green). n = 3 mice per group. **(C)** Representative images of microglia (green), CD68^+^ lysosomes (cyan), and postsynaptic marker Homer1 (red) in the striatum of WT, C3aR^-/-^, C3^-/-^, and astrocyte-specific knockout Aldh1l1^+/cre^; C3^fl/fl^ mice after 7-day infection by *S. aureus*. Scale bar = 10 μm. **(D)** Quantitation of Homer1 engulfment within microglial lysosomes. n = 35 cells in WT-SA group of three mice, n = 19 cells in C3aR^-/-^ SA group of three mice, n = 32 cells in C3^-/-^ SA group of three mice, n = 29 cells in Aldh1l1^+/cre^; C3^fl/fl^ SA group of three mice. **(E)** Representative images of immunostaining for Iba-1 (magenta), CD68 (red), and SA (green) in the striatum of WT, C3aR^-/-^, C3^-/-^, and astrocyte-specific knockout Aldh1l1^+/cre^; C3^fl/fl^ mice after 3-day infection by *S. aureus*. Scale bar = 15 μm. **(F)** Quantification of SA counts in the lysosomes (CD68) of microglia (Iba-1). n = 3 mice per group. Data are represented as mean ± SEM. One-way ANOVA with Dunnett’s multiple comparisons test for **(B)**, **(D)**, and **(F)** *p < 0.05, **p < 0.01, ***p < 0.001, and ****p < 0.0001.

To gain further insight into the functional rescue of synapse loss mediated by microglia phagocytosis in the context of C3aR or C3 ablation, we further examined synapse density by staining the presynaptic marker (Vglut1) and postsynaptic marker (Homer1) in the *S. aureus*-infected striatum. The co-localization of the presynaptic protein Vglut1 and postsynaptic protein Homer1 revealed a notably increased intensity in the C3-ablated, C3aR-knockout mice, or astrocyte-specific C3 conditional knockout mice as compared to the WT controls, respectively (Fig 7A and 7B).

To further investigate whether the restoration of lost synapses was associated with microglia-mediated synaptic engulfment, immunostaining of the postsynaptic marker (Homer1), lysosome marker (CD68), and microglial cell marker (Iba-1) were conducted. Using Imaris 3D imaging, the volume of Homer1-positive synapses within CD68-positive microglial lysosomes was measured. It was noted that a reduction in the presence of Homer1-positive synaptic puncta inside microglial lysosomes in the brains of mice infected with *S. aureus* was observed when C3 or C3aR was ablated, or astrocyte-specific C3 was conditionally knocked out (Fig 7C and 7D).

Furthermore, we examined *S. aureus* signals in microglial phagosomes to assess the impact of complement C3 and C3aR deficiency on the ability of microglia to phagocytize bacteria. We used immunostaining to label Iba-1, CD68, and *S. aureus* (SA) (Fig 7E). The number of *S. aureus* signals in the microglial lysosomes of the infected striatum on day 3 did not significantly differ from those in the Sham group (Fig 7F). Taken together, these findings signify that the genetic removal of C3-C3aR signaling significantly reinstates microglial phagocytosis-mediated synapse loss induced by *S. aureus* infection.

### Genetic deletion of complement C3 and C3aR reverses neuroinflammation and behavioral deficits in *S. aureus-*infected mice

**Fig 8:**
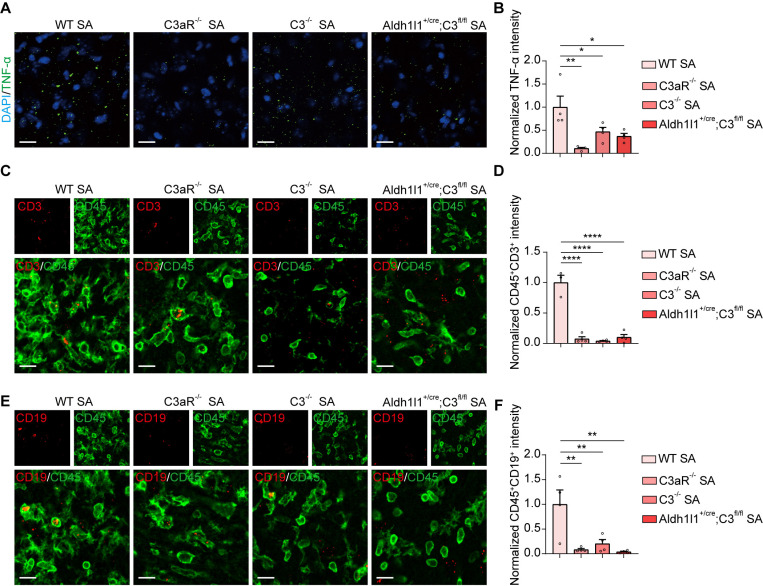
Deletion of C3 and C3aR attenuated *S. aureus-*induced inflammation. **(A)** Representative images of TNF-α (green) and DAPI (blue) in the striatum of WT, C3aR^-/-^, C3^-/-^, and astrocyte-specific knockout Aldh1l1^+/cre^; C3^fl/fl^ mice after 7-day infection by *S. aureus*. Scale bar = 15 μm. **(B)** Quantification of TNF-α signals intensity. n = 3 - 4 mice per group. **(C)** Representative images of CD3 (red) and CD45 (green) in the striatum of WT, C3aR^-/-^, C3^-/-^, and astrocyte-specific knockout Aldh1l1^+/cre^; C3^fl/fl^ mice after 7-day infection by *S. aureus*. Scale bar = 15 μm. **(D)** Quantification of CD3^+^CD45^+^signals intensity. n = 3 - 4 mice per group. **(E)** Representative images of CD19 (red) and CD45 (green) in the striatum of WT, C3aR^-/-^, C3^-/-^, and astrocyte-specific knockout Aldh1l1^+/cre^; C3^fl/fl^ mice after 7-day infection by *S. aureus*. Scale bar = 15 μm. **(F)** Quantification of CD19^+^CD45^+^ signals intensity. n = 3 - 4 mice per group. Data are represented as mean ± SEM. One-way ANOVA with Dunnett’s multiple comparisons test for **(B)**, **(D)**, and **(F)**, *p < 0.05, **p < 0.01, and ****p < 0.0001.

Bacterial meningitis is an inflammation of the meninges, primarily caused by bacteria that enter the central nervous system through the bloodstream, along with the neuroinflammatory responses triggered by the infection, ultimately leading to the death of neuronal cells [41]. We further investigated whether genetically removing C3-C3aR signaling contributes to the reduction of the neuroinflammatory response triggered by *S. aureus* infection. Through immunostaining, we observed a significant decrease in TNF-α levels in *S. aureus*-infected mice lacking C3 or C3aR (Fig 8A and 8B). Additionally, we performed co-staining with CD45 and CD3 to label T cells, and CD45 and CD19 to identify B cells (Fig 8C and 8E). Our findings showed that the deletion of C3 or C3aR resulted in a marked reduction in the colocalization of CD45^+^CD3^+^ and CD45^+^CD19^+^ signals (Fig 8D and 8F), indicating a decrease in T and B cell activity.

The pruning of synaptic material by microglia engulfment plays a crucial role in synapse and memory deficits in neurodegenerative diseases and virus infection [8,57–62]. Microglia are responsible for memories in a complement-dependent manner [49]. Given the improvement in synapse loss and the phagocytic features of microglia in *S. aureus*-infected C3 or C3aR ablated mice, we speculated that deleting C3 or C3aR could potentially restore memory deficits in *S. aureus*-infected mice.

**Fig 9:**
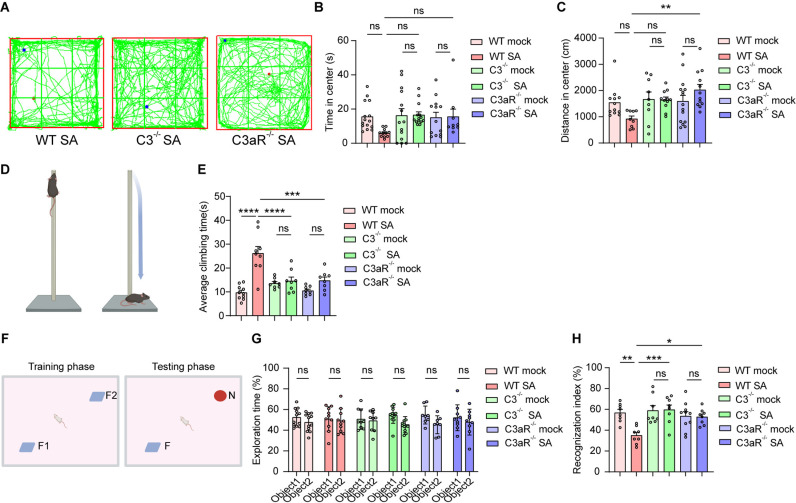
Deletion of C3 and C3aR attenuated *S. aureus-*induced cognitive impairment. **(A)** Representative images showed the trace of WT, C3^-/-^, and C3aR^-/-^ mice after 7-day infection by *S. aureus* in the open field test. **(B and C)** Center time (s) traveled (B) and center distance (cm) traveled (C) in the open field test. n = 8-12 mice per group. **(D)** Schematic diagram of pole test. Created with BioRender.com. **(E)** Climbing time of WT, C3^-/-^, and C3aR^-/-^ mice after 7-day infection by *S. aureus* in the pole test. n = 8-12 mice per group. **(F)** Schematic diagram of NOR test. Created with BioRender.com. **(G and H)** Exploration time (G) and Recognition index (H) of WT, C3^-/-^, and C3aR^-/-^ mice after 7-day infection by *S. aureus* in the NOR test. n = 8-12 mice per group. Data are represented as mean ± SEM. Two-way ANOVA with Tukey’s multiple comparison test for **(B and C)**, **(E)**, and **(G-H)**, *p < 0.05, **p < 0.01, ***p < 0.001, and ****p < 0.0001.

First, we found that the infection of *S. aureus* resulted in dramatic weight loss, which can be mitigated by the deletion of C3 (S4A Fig). The bacteria load was significantly reduced in the striatum of *S. aureus*-infected C3 or C3aR ablated mice (S4B Fig). We then investigate the exploratory or anxiety-like behaviors during the open-field test in C3^-/-^, C3aR^-/-^, and WT mice infected by *S. aureus*. We found that *S. aureus* infected C3^-/-^, and C3aR^-/-^ mice spent more time and distances in the center zone compared to mock-infected controls (Fig 9A–C). This increase in locomotion suggested that the deletion of C3 or C3aR could potentially alleviate anxiety and movement deficits. Additionally, the movement deficits in the WT mice infected by *S. aureus* were mitigated by the deletion of C3 or C3aR, as indicated by a reduced time observed in the pole test (Fig 9D–E). We employed the NOR testing to investigate the role of C3 or C3aR in this *S. aureus-*induced cognitive impairment. As expected, mice showed no preference for the two identical objects in the first phase, but *S. aureus*-infected mice showed no preference for the novel object in the second phase (Fig 9F–H). In contrast, *S. aureus*-infected C3^-/-^, and C3aR^-/-^ mice showed a significant preference for the novel object (Fig 9F–H). Overall, these findings suggested that C3-C3aR signaling plays a critical role in *S. aureus-*induced memory deficits and sickness-like behaviors.

## Discussion

**Fig 10:**
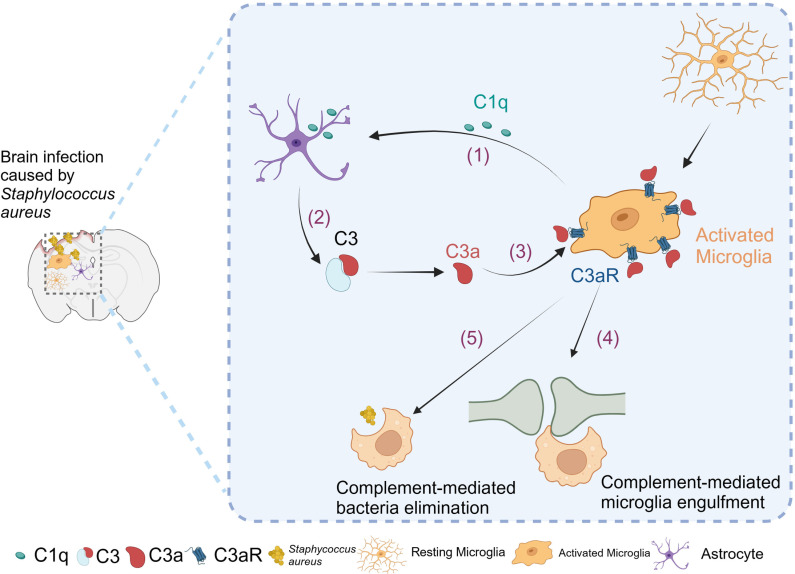
Schematic diagram showing that the complement C3-C3aR axis mediates microglial engulfment of synapses following brain infection caused by *S. aureus.* In brain infection caused by *S. aureus*, the following process might be triggered: (1) Upregulated C1q expression is released from activated microglia and acts on astrocytes. (2) Activation of astrocytes leads to the release of C3. (3) C3a activates microglia further through interaction with the C3aR. (4) The C3-C3aR axis facilitates the engulfment of synapses, but not bacteria, by microglia. Created with BioRender.com.

In this study, we identified that brain infection caused by *S. aureus* can activate microglia to phagocytosis synapses mediated by the C3-C3aR axis (Fig 10). In the context of brain infection in wild-type mice, we observed neuronal damage, reduced synaptic density, cognitive impairment, and motor dysfunction. Notably, when the C3-C3aR axis was genetically blocked, a reduction in microglial phagocytic activity was observed, coupled with an improvement in synapse loss, inflammatory response, cognitive impairment, and sickness behaviors.

Bacteria have the capability to enter the central nervous system via the bloodstream and cross the blood-brain barrier (BBB). Once they penetrate the BBB, these bacterial pathogens interact with neurons and glial cells in the brain through the release of toxins or direct contact. This interaction, along with the neuroinflammatory responses triggered by the infection, can ultimately lead to the death of neuronal cells [41]. Furthermore, even if neurons remain structurally intact, the infection may disrupt neuronal signaling pathways and neuroinflammatory responses, thereby affecting synaptic activity. It’s important to recognize that infections caused by microbes, including both viruses and bacteria, can significantly impact cognitive function [63,64]. Infection with West Nile virus (WNV) or Zika virus (ZIKV) has been associated with an impact on hippocampal-dependent learning and memory [61]. Individuals with sepsis may encounter challenges related to cognition, memory, and sensory processing [65,66]. Likewise, patients with brain infections caused by gut microorganisms may also experience cognitive impairments [67–69]. The loss of synapses is identified as a crucial contributor to cognitive impairment [70]. In our study, the behavioral studies and immunostaining further corroborated the memory impairment, inflammatory response, neuronal damage, and synapse loss in the acute and late stages of *S. aureus* infections. Our research indicates that neuronal damage occurs not only in the infected regions but also in remote regions, such as the hippocampus. Notably, we have identified that the toxin released by *S. aureus* can induce cell death, a process that can be ameliorated by the deletion of the α-toxin. Additionally, our findings show clear evidence of the adhesion and internalization of *S. aureus* by neurons, supporting and expanding upon previous studies regarding neuronal death in pneumococcal meningitis [42]. This highlights the potential for targeted interventions to mitigate neuronal damage in these cases.

Microglia serve as the brain’s primary phagocytic cells, and they play a crucial role in refining synaptic circuits and protecting the brain by phagocytosing bacteria, abnormal proteins, and cellular debris [71]. During normal development, microglia shape neuronal circuits by removing excess synapses, dendrites, axons, myelin sheaths, and neurons [30]. In response to substantial injury or pathogen attack, microglia transition from a resting state to an amoeboid state and release pro-inflammatory factors and cytokines [38]. Insightful knowledge about the physiological and pathological properties of microglia has been accumulated through *in vivo* and *in vitro* studies involving neurotropic viruses, bacteria, fungi, parasites, and prions. During the recovery from Zika virus or West Nile virus infection, sustained T cell production of interferon (IFN)-γ may trigger microglial activation [61]. In the context of *S. aureus* infections in the brain, there was a notable increase in gliogenesis, specifically affecting microglia and astrocytes [72,73]. This heightened gliogenesis coincided with the onset of the infections. Additionally, reactive microglia increased their phagocytosis of synapse puncta and bacteria, along with non-professional phagocytes like astrocytes in their phagocytosis of bacteria over the course of the *S. aureus* infections. These studies have yielded new perspectives, shedding light on the intricate and remarkably complex interactions between microglia, astrocytes, and neurons underlying central nervous system infections [74].

The complement system serves as a vital component of the innate immune system and contributes to the maintenance of central nervous system functions. However, abnormal activation of the complement system has been associated with various central nervous system diseases, such as stroke, traumatic brain injury, multiple sclerosis, and Zika virus infection. During normal development, C1q and C3 are necessary for synaptic pruning to refine synaptic circuits [31,35]. Conversely, excessive pruning by the complement system during pathological states may result in unwarranted synaptic elimination, and this has been associated with cognitive deficits [57,75]. Bacterial pathogenesis is strongly influenced by the strength of the host’s immune defense. In a previous study, it was observed that in C57BL/6 mice infected with *S. aureus*, a considerable number of upregulated host genes were found to be associated with the KEGG pathways related to ‘cytokine-cytokine receptor interaction’, ‘chemokine signaling pathway’, and ‘complement and coagulation cascades’. Specifically, inflammatory cytokines such as IL-6, IL-1α, IL-1β, and TNF-α, as well as chemokines that attract monocytes/macrophages, including Cxcl1, Cxcl2, and Cxcl3, were observed to be upregulated in response to the *S. aureus* infection in C57BL/6 mice kidney [76]. Through analysis of the transcriptional profiles of *S. aureus-*infected microglia in the mouse brain, we demonstrate that a few gene sets of inflammation-related genes involved in the TNF signaling pathway, NF-κB signaling pathway, Toll-like receptor signaling pathway, and phagosome were highly expressed in response to the early stage of *S. aureus* infections. Recurrent *S. aureus* infections elicited immune cell responses in mice that expressed the major histocompatibility complex (MHC), a part of antigen processing and presentation [77]. In this study, we also observed the enrichment of gene sets related to antigen processing and presentation, complement and coagulation cascades, and NF-κB signaling pathways in the *S. aureus*-infected microglia. Our findings revealed a remarkable increase of complement proteins C1q, C3, and C3aR in the *S. aureus*-infected brain lysate, indicating an activation of the complement system induced by *S. aureus* infections.

The conventional understanding was that the liver was the primary producer of complement proteins. However, recent research has uncovered that complement proteins can be generated by astrocytes, microglia, neurons, and other cells in the central nervous system. In cell culture studies, primary cultures of mouse and rat astrocytes consistently express C3 mRNA and produce C3 protein [78,79]. Both the mRNA and protein levels of C3 were significantly enhanced by LPS or by a live and inactivated Newcastle disease virus [78,80]. Research using qRT-PCR detected C1q, C2, C3, and C4 mRNA in microglial cultures, while C3 and C4 mRNA were found in astrocyte cultures [81]. Additionally, IL-1β increases the binding capacity of p65 to the NF2 site of the C3 promoter, promoting C3 expression in rat primary astrocytes and microglia [82]. In *in vivo* studies, native C3 is upregulated in astrocytes of APP mice [83], consistent with other research showing increased C3 synthesis in cultured astrocytes after injury. Significant increases in genes related to classical pathway activation (C1q to C3) were observed in sorted astrocytes from TauP301S and PS2APP mice, while genes for other complement pathways showed no change. Moreover, microglia from PS2APP mice did not exhibit this change. Neurons displayed low expression of complement genes without significant alteration in these models [51]. Additionally, complement C3 primarily colocalized with astrocytes in the spinal dorsal horn of a rat chronic constriction injury model [84]. RNA sequencing revealed upregulation of C3 in reactive GLAST^+^ astrocytes from laparotomy [85], and increased levels were also noted in astrocytes from both a pilocarpine-induced status epilepticus model [86], and traumatic brain injury brain [87]. Our studies indicated that complement C3 was primarily derived from astrocytes in the *S. aureus*-infected brain. Therefore, to further understand the role of complement C3 and associated signaling, we employed C3 or C3aR knockout mice or astrocyte-specific C3 conditional knockout mice. The reduced synapses, increased complement activation, enhanced microglial phagocytosis, and cognitive impairment were all alleviated in mice lacking C3, the C3a receptor, or with astrocyte-specific C3 gene deletion, compared to WT controls. These results align with the observation that deficiencies in complement C3 or C3aR protect against WNV-induced synaptic terminal loss [57]. These findings imply that the removal of C3-C3aR signaling significantly restores microglial engulfment of synaptic material, prevents synapse loss, and reverses behavioral impairment induced by *S. aureus* infection. However, we did not observe a reduction in bacterial phagocytosis after the deletion of complement C3 or C3aR. This insight suggests that microglial phagocytosis of bacteria might function independently of C3-C3aR signaling, paving the way for further investigation into alternative pathways and mechanisms involved. This work has several potentially important implications for *S. aureus*-induced infection. Our data suggests a significant role for microglial C3aR and astrocytic C3 in defending against *S. aureus*. Additionally, it appears that C3aR-independent actions of microglia may have a contributory effect on neuroinflammation by facilitating astrocyte recruitment to the sites of infection. This work still has some limitations. For example, the nearly ubiquitous inflammatory response of *S. aureus* raises the question of whether other cells in the CNS may contribute to the neuroinflammation from infection and/or colonization. MACS enrichment of CD11b^+^ cells from inflamed brain tissue may include infiltrating immune cells (e.g., monocytes, macrophages, or neutrophils) alongside resident microglia, as CD11b is expressed by both populations. This approach cannot entirely exclude non-microglial cells, particularly under inflammatory conditions. The RNA sequencing on isolated CD11b^+^ cells at D1 and D7 post-infection through MACS analysis cannot exclude the effects of other CD11b^+^ myeloid cells. Future studies could benefit from implementing more rigorous purification techniques followed by additional fluorescence-activated cell sorting to remove other infiltrating CD11b^+^ immune cells through the expression of the leukocyte antigen CD45, which could further refine the isolation process and improve the quality of the research findings. Nonetheless, our findings regarding microglial activation and associated inflammation, indicated by the amoeboid morphology of microglia in our staining, are both compelling and noteworthy. Second, the removal of C3-C3aR signaling alleviates astrocyte-microglia crosstalk is intriguing, but the impact of microglia-inherent variability influences the transcriptional response of *S. aureus* during *in vivo* infection is likely to be limited. Last, it would not be anticipated that the expression of virulence factors by *S. aureus* is determinant to microglial immune response, facilitating the recruitment of astrocytes in the infection.

## Materials and methods

### Ethics statement

The animal protocols for the experiments in this study were approved by the Ethical Committee of Soochow University.

### Bacterial strains and cultures

The *S. aureus* strain (USA300) was securely maintained at the laboratory of the Second Affiliated Hospital of Soochow University. The *S. aureus* strains (Newman, Newman Δ*hla,* Newman Δ*hla*+pC*hla*) were graciously provided by Professor Xiancai Rao from the Army Medical University. The Newman, Newman- Δ*hla* strain underwent a meticulous culturing process in tryptic soy broth (TSB) at 37 °C with gentle shaking at 200 rpm overnight, followed by a precise 1:100 dilution in TSB for subsequent cultivation. Similarly, the Newman Δ*hla*+pC*hla* strain was cultured overnight at 37 °C in TSB containing 10 µg/mL of chloramphenicol, also with gentle shaking at 200 rpm. Afterward, a 1:100 dilution in TSB containing 10 µg/mL chloramphenicol was prepared for subsequent cultivation.

### Animals

C57BL/6 mice (8–9 weeks of age) were purchased from the Suzhou SinoCell Biotechnology Co., Ltd. (Suzhou, China). B6.129S4-C3^tm1Crr^/J (C3^−/−^, Strain #:003641) mice obtained from the Jackson Laboratory were gifted by Dr. Weiguo Hu (Fudan University). C57BL/6JGpt-C3ar1^em6Cd4816^/Gpt (C3aR^−/−^, strain NO. T006106), C57BL/6JGpt-C3^em1Cflox^/Gpt (C3^flox/flox^, NO. T009469), C57BL/6JGpt-Aldh1l1^em1Cin(CreETR2)^/Gpt (Aldh1l1^CreERT2^, NO. T052693) mice generated from Gempharmatech Biotechnology (Nanjing, China) were gifted by Dr. Chao Yan (Nanjing University). These genetic mouse strains were bred in a specific pathogen-free animal facility of Soochow University (Temperature 22 °C, humidity 59 rH using a 12/12 h dark/light cycle).

### *S. aureus*-induced mouse brain infection model

For the procedure, female C57BL/6 mice were appropriately anesthetized with Avertin (0.025 mL per g) [88] and gently secured in a stereotactic frame (RWD, Life Science Co, Shenzhen, China). Agarose beads encapsulated with *S. aureus*, as the previously established protocol [39], were meticulously prepared, with agarose beads encapsulated with PBS utilized as the control. A suspension of 5 μL of encapsulated *S. aureus*-encapsulated agarose beads (1 × 10^5^ CFU) was carefully injected into the right striatum at the specified coordinates: 0.8 mm rostral, 2 mm lateral to the right of bregma, and 3.0 mm ventral to the bregma. As a sham control, pure agarose beads were also injected. To avoid any potential reflux, the needle was maintained in place for 5 minutes. Following the needle withdrawal, the scalp was sutured. After the completion of the infection procedure, the mouse was successfully restored and returned to its original home cage. The weights of the mice were routinely recorded every other day to monitor their growth and health. The animal behavior study was conducted on D7, D14, and D28 after the *S. aureus* infection.

### Behavioral tests

Open field test. We evaluated locomotor activity using an acrylic box (40 cm × 40 cm × 40 cm, L × W × H) for the open field test. Each mouse was placed in the same corner of the box to explore the environment for 10 minutes. Data was automatically recorded and analyzed by Supermaze (XR-XZ301, Shanghai XinRuan Information Technology Co., Ltd., China) to quantify exploratory behavior.

Novel object recognition. The test was carried out in an acrylic box (40 cm × 40 cm × 40 cm, L × W × H), where wooden objects of different shapes and colors were affixed using double-sided adhesive tape. Objects were strategically placed along the diagonal at a certain distance from the wall, with the mouse positioned in the center of the diagonal. Prior to the test, each mouse received a 30-minute training session in a spacious and clean box.

During the training stage, two objects of identical color and shape were placed in the box. Each mouse was freely explored for 10 minutes and the time spent exploring each object was recorded. To ensure the elimination of the olfactory cures, the box and objects were cleaned with 70% ethanol between trials. Two hours later, the mice were reintroduced to the box for the test session, with one of the training objects being replaced by a novel object. The duration of exploration for each object was recorded. Results are presented as the percentage of time spent exploring each object during the training or testing stage. Data was automatically recorded and analyzed by video tracking software (XR-XZ301, Shanghai XinRuan Information Technology Co., Ltd., China).

Pole Test. The experimental apparatus is comprised of a 50-cm-high and 0.5-cm-diameter wooden pole, which is wrapped with gauze at the top as previously described [89]. During the training phase, mice were placed at the top of the pole and allowed to freely climb down three times. Subsequently, in the testing phase, the time taken for mice to descend from the top of the pole was meticulously recorded three times.

### Western blot

The striatum region was efficiently homogenized in a lysis buffer containing phosphatase and protease inhibitors. The protein extracts were then carefully loaded on 7.5%/10%/12.5% polyacrylamide gels and subjected to electrophoresis at 80V to concentrate the gel and separate the gel boundary, followed by electrophoresis at 120V to effectively separate the protein bands. The proteins were subsequently transferred onto a PVDF membrane, blocked in 5% skim milk, and incubated overnight at 4 °C with the specified primary antibodies: rabbit anti-C1q (Abclonal, A24519, 1:1000), Goat anti-C3 (MP, 855730–2ML, 1:1000), rabbit anti-C3aR (Abclonal, A6361, 1:1000), mouse anti-GAPDH (FUDE, FD0063, 1:1000), mouse anti-β-actin (FUDE, FD0060, 1:1000), mouse anti-β-tubulin (FUDE, FD0064, 1:1000). Membranes were washed three times for 15 minutes each with TBST. Membranes were incubated for 2 hours at room temperature with the following secondary antibodies: goat anti-rabbit HRP (FUDE, FDR007), goat anti-mouse HRP (FUDE, FDM007), donkey anti-goat IgG(H+L) (Jackson ImmunoResearch, 705-035-003). Protein bands were visualized using an enhanced chemiluminescence detection kit (New cell & Molecular Biotech, P10300). The intensity of each band was analyzed using ImageJ.

### Cell culture

The primary hippocampal neuronal culture was prepared from C57BL/6J pups at postnatal day 0 (P0) after anesthetizing and decapitating them. The hippocampal tissue was dissected in a dissection medium (DM) containing 1 mM sodium pyruvate, 0.2% glucose, 1% Penicillin-Streptomycin, 10 mM HEPES, pH 7.4 in HBSS-Ca/Mg free. To dissociate the cells, the tissue was treated with 0.1 mg/mL Papain and 0.2 mg/mL DNase I in DM for 15 minutes at 37 °C, followed by wash 2 times with stop medium (DM with 20% FBS) and centrifugation for 5 min under 350 × g. The neurons were resuspended in a culture medium (Neurobasal-A with 2% B-27, 1% GlutaMAX, 5% FBS, and 1% Penicillin-Streptomycin) and counted. Neurons were plated on poly-D-lysine coated glass-bottom dishes in a 24-well plate at a density of 0.3 × 10^6^. After 4 hours, 80% of the medium was refreshed, with additional exchanges on day 3 and day 7 with fresh Neurobasal-A supplemented with 2% B-27, 1% GlutaMAX, 2.5% FBS, 50 μM FuDR, and 1% Penicillin-Streptomycin. By day 10, neurons were again refreshed about 80% of the culture medium using Neurobasal-A with 2% B-27, 1% GlutaMAX, and 1% Penicillin-Streptomycin. Two days later, the primary neurons cultured in Neurobasal-A with 2% B-27, 1% GlutaMAX were prepared for bacterial invasion experiments. The invasion MOI (Multiplicity of infection) of *S. aureus* is 5:1. The invasion time is 2 hours. Then wash three times with PBS and fix with 4% PFA for half an hour for subsequent immunofluorescence. The mouse BV2 microglia cell line was obtained from the Institute of Neuroscience at Soochow University. The BV2 cell line was cultured in high-glucose DMEM (SH30243.01, HyClone, USA) containing 10% FBS (16000-044, Gibco). The invasion MOI of *S. aureus* is 5:1. The invasion time is 2 hours. The C6 glioma cell line was obtained from the Institute of Neuroscience at Soochow University. The C6 cell line was cultured in Ham’s F-12K (10-080-CV, CORNING) containing 2.5% FBS (16000-044, Gibco) and 15% Horse Serum (BL209A, Biosharp). The invasion MOI of *S. aureus* is 5:1. The invasion time is 2 hours.

### Immunostaining

Mice were anesthetized with Avertin (0.025 mL/g) and perfused with PBS and 4% paraformaldehyde (PFA). After this, leave the brains in 4%PFA overnight and cryoprotect them in a 30% sucrose solution for two days. Slice the brains into coronal sections of 30 μm thickness. Wash slices first in PBS for 20 minutes three times, then in PBST (0.025% Triton X-100) for 20 minutes, repeating the cycle three times. After that, block the slices by incubating them in either 5% goat serum or a combination of 5% goat serum and 5% donkey serum for one hour. Following this, incubate the slices with primary antibodies overnight. Wash slices first in PBST for 20 minutes three times, then incubated with second antibodies for two hours. Image using LSM700, LSM800 or LSM900 laser-scanning confocal microscope (Carl Zeiss, Germany) dependent on different assays. The primary antibodies were as follows: Goat anti-C3 (MP, 855730-2ML, 1:1000), Guinea pig anti-Vglut1 (Synaptic Systems, 135-304, 1:500), chicken anti-Homer1 (Synaptic Systems, 160006, 1:500), rabbit anti-Iba-1 (Wake Chemicals, 019-19741, 1:500), Guinea pig anti-GFAP (Oasis bioform, OB-PGP055-02, 1:1000), rabbit anti-C1q (Abclonal, A24519, 1:250), chicken anti-Iba-1 (Synaptic Systems, 234009, 1:1000), rat anti-CD68 (Abcam, ab53444, 1:500), chicken anti-NeuN (Merck, ABN91,1:500), rabbit anti-MAP2 (ThermoFisher Scientific, PA5-17646, 1:100), mouse IgG1 anti-Caspase3 (ThermoFisher Scientific, 43-7800,1:250), mouse IgG1 anti-NeuN (Proteintech, 66836-1-lg, 1:250), rabbit anti-*S. aureus* (Abcam, ab20920,1:1000), rat anti-LAMP2 (Abcam, ab13524,1;500), Guinea pig anti-Iba-1 (Oasis bioform, OB-PGP049-01, 1:1000), rabbit anti-TNF-α (Abclonal, A11534, 1:100), rabbit anti-CD45 (Proteintech, No.83396-5-RR, 1:250), mouse anti-CD19 (Proteintech, No. 66298-1-Ig, 1:250), mouse anti-CD3 (Beyotime, AG1383, 1:50). The second antibodies were as follows: goat anti-rat IgG(H+L) Alexa Fluor 647 (Cell signaling technology, 4418, 1:500), goat anti-chicken IgY Alexa Fluor 555 (Abcam, ab150170, 1:500), goat anti-rabbit IgG Alexa Fluor 488 (AIFANG, AFSA005, 1:200), goat anti-rabbit IgG Alexa Fluor 647 (Abcam, ab150083, 1:500), goat anti-mouse IgG (H+L) Alexa Fluor 488 (Abcam, 4408S, 1:500), goat anti-rabbit IgG Alexa Fluor 488 (Abcam, ab150077, 1:500), goat anti-Guinea pig Alexa Fluor 647 (Oasis bioform, GP647, 1:500), donkey anti-goat Alexa Fluor 488 (AIFANG, AS032, 1:200), goat anti-Guinea pig Alexa Fluor 555 (ThermoFisher Scientific, A21435, 1:500), goat anti-Guinea pig Alexa Fluor 488 (Abcam, ab150185, 1:500), goat anti mouse IgG1 CY5 (Abcam, AB136127, 1:500). All sections were stained with DAPI (ThermoFisher Scientific, D1306, 1:500).

### Microglial phagocytosis of synapses

The process of quantifying Homer1 engulfment within microglia in mice involved utilizing Imaris Reconstruction software (v.9.9, Bitplane) according to previously described methods [31,90]. High-resolution confocal images of Iba-1^+^ cells with CD68 and Homer1 staining were used for reconstructions. The image processing tool in Imaris was utilized to perform background subtraction and a Gaussian filter. Iba-1^+^ cells were surface rendered with 0.1-μm smoothing, and disconnected processes were combined with the cell body to create a single surface. A mask was applied to the channel containing CD68 to isolate the CD68 signal exclusively within the microglia for surface rendering (0.1-μm smoothing). For Homer1 engulfment assays, a mask was applied to the Homer1 channels to showcase only the Homer1 signal within microglial lysosomes. The isolated Homer1 channels were surfaced and rendered (0.1-μm smoothing) to record the volume. The percentage engulfment of Homer1 within microglial lysosomes was calculated using the formula: volume of engulfed material within lysosome/volume of Iba-1^+^ cell.

### Microglial and astrocytic phagocytosis of *S. aureus*

High-resolution confocal images of Iba-1^+^ cells with CD68 and *S. aureus* (SA) staining and confocal images of GFAP^+^ cells with LAMP2 and *S. aureus* (SA) were used for quantifying *S. aureus* engulfment within microglia and astrocytes in mice, respectively. The percentage engulfment of SA counts within microglial and astrocytic lysosomes was calculated using the formula: counts of engulfed SA signals within Iba-1^+^ lysosome or GFAP^+^ lysosome.

### Isolation of microglia and RNA sequencing of microglia against *S. aureus* infection

The mice were gently anesthetized with Avertin (0.025 mL/g) and then perfused with PBS. After removing meninges and blood vessels, the striatum was carefully isolated and dissociated into a single cell suspension using 0.05% trypsin (S310KJ, BasalMedia, Shanghai). CD11b Microbeads (130-093-636, Miltenyi Biotec) were utilized to label CD11b-positive microglia and then loaded onto a MACS Column (Miltenyi Biotec) for magnetic separation. Isolated microglia were then extracted total RNA by TRIzol reagent (Invitrogen, USA). Ribosomal RNA (rRNA) was conscientiously removed from the samples using the GenSeq rRNA Removal Kit (GenSeq, Inc.). Following the rRNA removal, the samples were meticulously utilized to construct sequencing libraries using the GenSeq Low Input RNA Library Prep Kit (GenSeq, Inc.). The constructed sequencing libraries were carefully subjected to quality control and quantification by BioAnalyzer 2100 system (Agilent Technologies, USA), followed by 150 bp double-end sequencing using IlluminaNovaSeq 6000 instrument. The raw data were obtained after sequencing on IlluminaNovaSeq 6000 instrument. Raw data quality control was first performed using Q30 values. Splices were de-joined using cutadapt (v1.9.3) to remove low-quality reads and high-quality cleanreads. Cleadreads were aligned to the reference genome using hisat2. Then, HTSeq (v0.9.1) was used to obtain raw count numbers, which were normalized using edgeR and ploidy change, and the p-value was calculated between the two sets of samples to screen for differentially expressed genes. GO functional, KEGG pathway, and GSEA analysis were performed using differentially expressed genes.

### Isolation of adult microglia and astrocytes for *S. aureus* infection, followed by ELISA test

The mice were gently anesthetized with Avertin (0.025 mL/g) and then perfused with PBS. Following the removal of meninges and blood vessels, the striatum was carefully isolated and dissociated into a single-cell suspension using a 70 µm cell strainer. The single-cell suspension was prepared for isolating microglia cells (Anti-CD11b MicroBeads, Miltenyi Biotec) and astrocytes (Anti-ACSA-2 MicroBeads, Miltenyi Biotec) according to the manufacturer’s instructions. Furthermore, microglia were labeled with CD11b-FITC (F41011b01, liankebio) and CD45-APC (F2104503F41011b01, liankebio) antibodies for 15 min at room temperature, and CD45^low^CD11b^+^ cells were identified by BD FACSVeres Flow Cytometer (BD, USA). Intracellular proteins were released through repeated freeze-thaw cycles using liquid nitrogen and a 37 °C water bath three times. The supernatant was collected after centrifugation at 2500 rpm for 20 minutes. C3 levels were measured using an ELISA kit (LV30768, Animalunion Biotechnology Co., Ltd) according to the manufacturer’s instructions.

### Bacterial load determination

The mice were euthanized on Day 7 following infection with *S. aureus*. The striatum was harvested, weighed, and homogenized in PBS. The homogenate was then serially diluted and plated onto TSB agar plates. After that, the plates were incubated at 37 °C for 24 hours to allow for accurate counting of the bacterial colonies.

### Statistical analysis

GraphPad Prism V.8 was applied for all statistical analyses. All data are presented as the mean ± SEM. An unpaired two-tailed Student’s t-test or paired two-tailed Student’s t-test was utilized to assess the differences between the two groups depending on the specific assay. In instances involving more than two groups, one-way ANOVA followed by Dunnett’s multiple comparisons test or Tukey’s multiple comparisons test and two-way ANOVA followed by Tukey’s multiple comparisons test or Šídák’s multiple comparisons test to compare every mean with every other mean were employed.

## Supporting information

S1 Fig Microglia activation in the contralateral striatum against *S. aureus* infection. (A) Representative images of Iba-1 (green) and CD68 (red) in the contralateral striatum from C57BL/6 mice injected with PBS (Sham) and C57BL/6 mice infected with *S. aureus* on day 3 (D3). Scale bar = 15 μm. (B) Quantification of CD68^+^Iba-1^+^ signals intensity. n = 4 mice per group. Data are represented as mean ± SEM. Unpaired Student’s t-test for (B), **p < 0.01.
(TIF)

S2 Fig Immune response and classical complement activation were involved in microglia after *S. aureus* infection. (A and B) GO enrichment analysis in the biological process of upregulated gene expression on D1 (A) and D7 (B) after *S. aureus* infection compared to that of the Sham group. (C and D) Immunoblotting analysis (C) and quantification (D) of the expression of complement proteins C3aR, C3 α-chain, and C1q. n = 3 mice per group. (E and F) Representative immunostaining images (E) and quantification (F) of the expression of complement proteins C1q. n = 3–5 mice per group. Data are represented as mean ± SEM. One-way ANOVA with Dunnett’s multiple comparisons test for (D) and (F), *p < 0.05, **p < 0.01, and ***p < 0.001.
(TIF)

S3 Fig Classical complement activation in astrocyte C6 and microglia BV2 cells against *S. aureus* infection. (A) Immunoblotting analysis of the expression of C3 α-chain in astrocyte C6 cells after *S. aureus* treatment at 0, 2, 4, and 6 h. (B) Quantification of C3 α-chain expression. n = 3 replicates per group. (C) Representative images of C1q (green), Iba-1 (red), and DAPI (blue) in the striatum from C57BL/6 mice injected with PBS (Sham) and C57BL/6 mice infected with *S. aureus* on day 3 (D3) and day 7 (D7). Scale bar = 15 μm. (D) Quantification of C1q^+^Iba-1^+^ signal intensity. n = 3–4 mice per group. Data are represented as mean ± SEM. One-way ANOVA with Dunnett’s multiple comparisons test for (B) and (D), *p < 0.05.
(TIF)

S4 Fig The impact of weight loss and bacterial load in mice deficient in C3 and/or C3aR induced by *S. aureus.* (A) The percentage change in body weight loss of WT and C3^-/-^ mice over time. n = 7–12 mice per group. (B) Bacterial load in the striatum of WT, C3^-/-^, and C3aR^-/-^ mice after 7-day infection by *S. aureus*. n = 4–8 mice per group. Data are represented as mean ± SEM. Two-way ANOVA with Tukey’s multiple-comparison test for (A). WT SA vs WT mock, *p < 0.05, ***p < 0.001, and ****p < 0.0001. C3^-/-^ SA vs WT SA, ^#^p < 0.05. One-way ANOVA with Dunnett’s multiple comparisons test for (B). **p < 0.01 and ****p < 0.0001.
(TIF)

S1 Table Differential gene expression (DEG), GO and KEGG analysis for upregulated DEG in microglia. (A) Upregulated DEGs on D1 after *S. aureus* infection compared to that of the Sham group. (B) Downregulated DEGs on D1 after *S. aureus* infection compared to that of the Sham group. (C) GO enrichment analysis of upregulated gene expression on D1 after *S. aureus* infection compared to that of the Sham group. (D) KEGG enrichment analysis of upregulated gene expression on D1 after *S. aureus* infection compared to that of the Sham group. (E) Upregulated DEGs on D7 after *S. aureus* infection compared to that of the Sham group. (F) Downregulated DEGs on D7 after *S. aureus* infection compared to that of the Sham group. (G) GO enrichment analysis of upregulated gene expression on D7 after *S. aureus* infection compared to that of the Sham group. (H) KEGG enrichment analysis of upregulated gene expression on D7 after *S. aureus* infection compared to that of the Sham group.
(XLSX)
