# Supplementary figures and images for: Astrocyte-derived complement C3 facilitated microglial phagocytosis of synapses in Staphylococcus aureus-associated neurocognitive deficits
Source: PLoS Pathog. 2025 Apr 28;21(4):e1013126. doi: 10.1371/journal.ppat.1013126 (PMC12121917; doi:10.1371/journal.ppat.1013126)

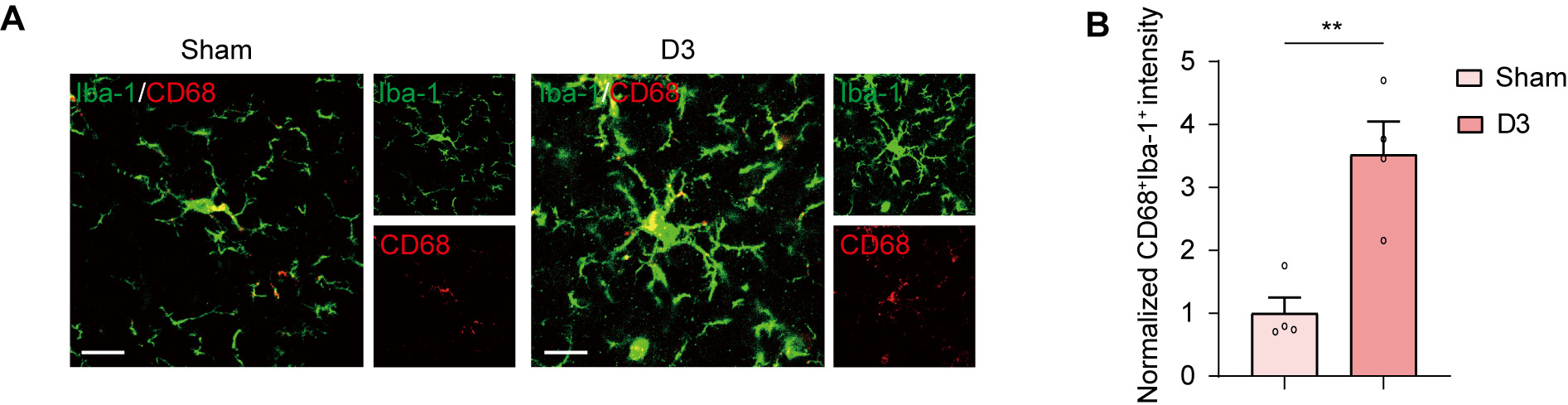

Supplement: S1 Fig — (A) Representative images of Iba-1 (green) and CD68 (red) in the contralateral striatum from C57BL/6 mice injected with PBS (Sham) and C57BL/6 mice infected with S. aureus on day 3 (D3). Scale bar = 15 μm. (B) Quantification of CD68+Iba-1+ signals intensity. n = 4 mice per group. Data are represented as mean ± SEM. Unpaired Student’s t-test for (B), **p < 0.01. (TIF) [file ppat.1013126.s001.tif]

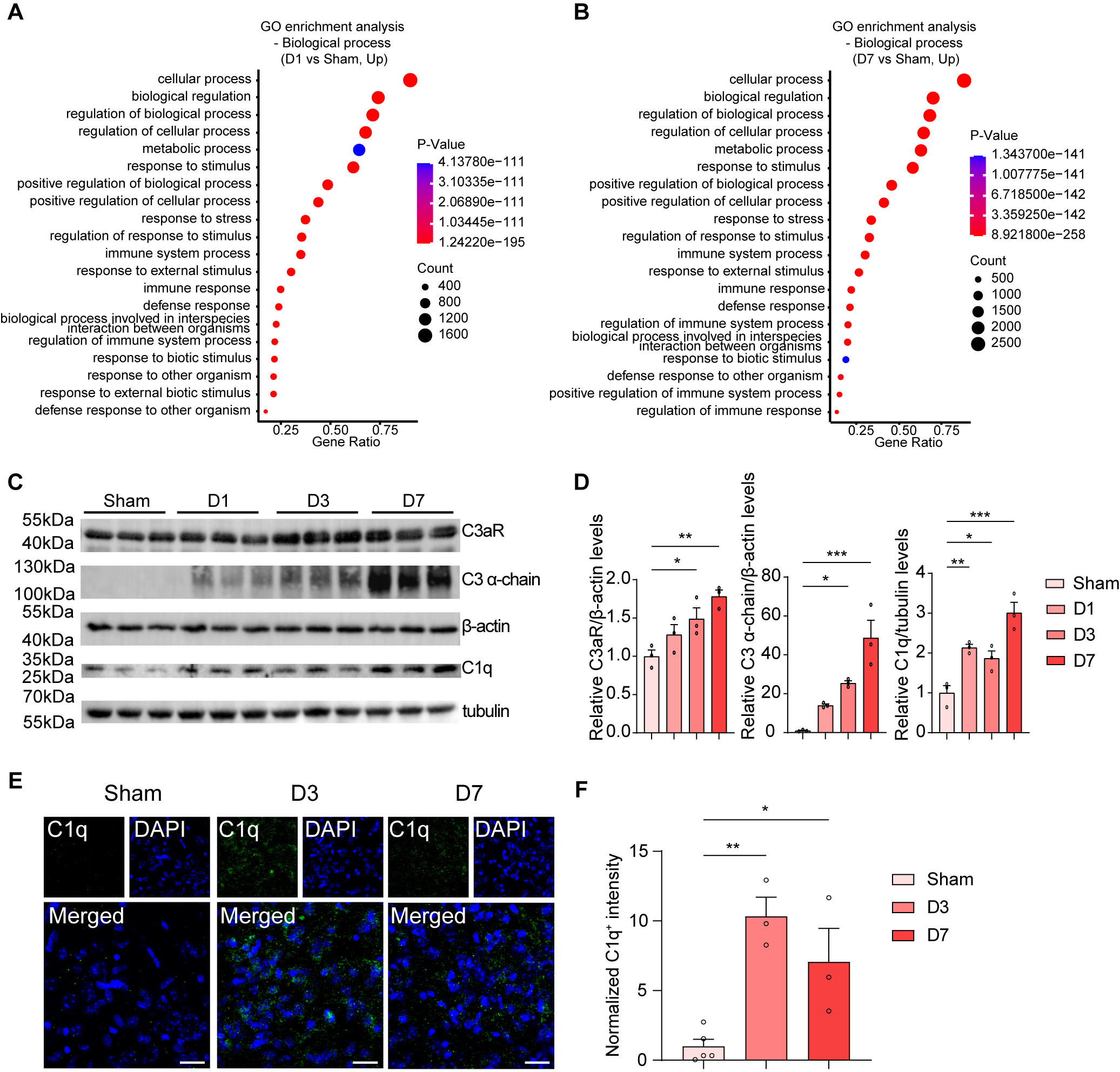

Supplement: S2 Fig — (A and B) GO enrichment analysis in the biological process of upregulated gene expression on D1 (A) and D7 (B) after S. aureus infection compared to that of the Sham group. (C and D) Immunoblotting analysis (C) and quantification (D) of the expression of complement proteins C3aR, C3 α-chain, and C1q. n = 3 mice per group. (E and F) Representative immunostaining images (E) and quantification (F) of the expression of complement proteins C1q. n = 3–5 mice per group. Data are represented as mean ± SEM. One-way ANOVA with Dunnett’s multiple comparisons test for (D) and (F), *p < 0.05, **p < 0.01, and ***p < 0.001. (TIF) [file ppat.1013126.s002.tif]

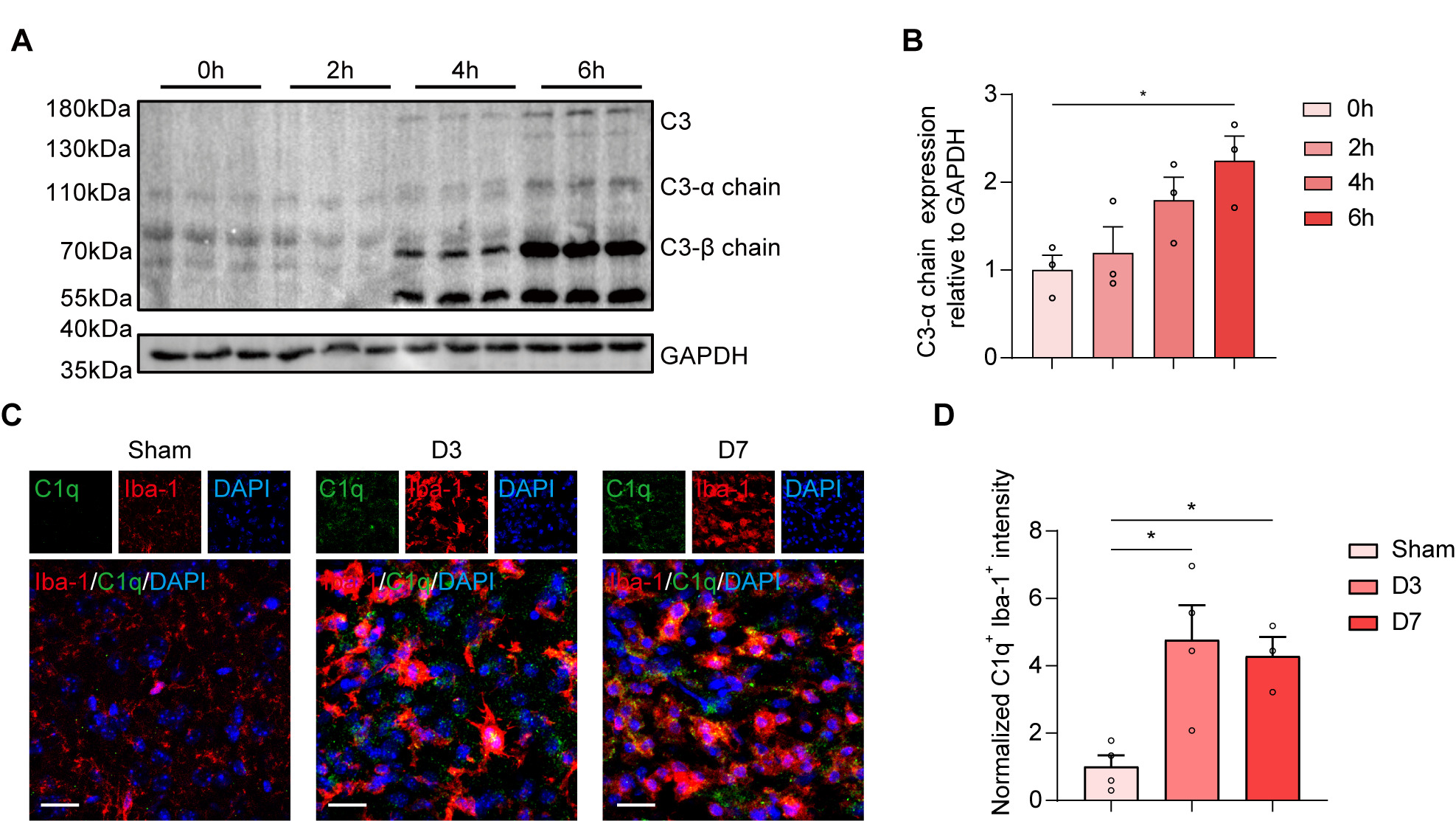

Supplement: S3 Fig — (A) Immunoblotting analysis of the expression of C3 α-chain in astrocyte C6 cells after S. aureus treatment at 0, 2, 4, and 6 h. (B) Quantification of C3 α-chain expression. n = 3 replicates per group. (C) Representative images of C1q (green), Iba-1 (red), and DAPI (blue) in the striatum from C57BL/6 mice injected with PBS (Sham) and C57BL/6 mice infected with S. aureus on day 3 (D3) and day 7 (D7). Scale bar = 15 μm. (D) Quantification of C1q+Iba-1+ signal intensity. n = 3–4 mice per group. Data are represented as mean ± SEM. One-way ANOVA with Dunnett’s multiple comparisons test for (B) and (D), *p < 0.05. (TIF) [file ppat.1013126.s003.tif]

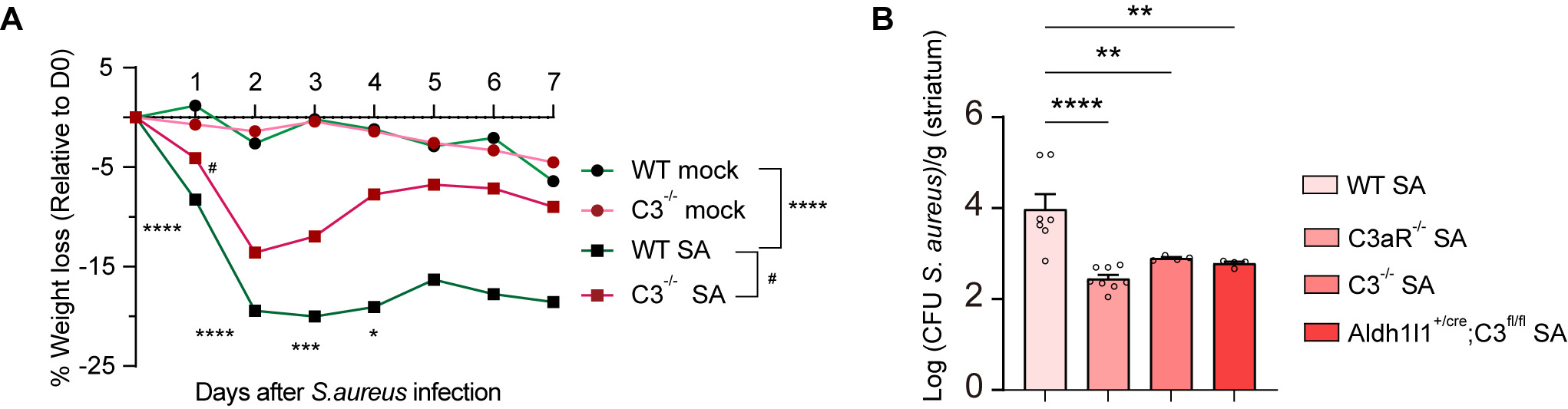

Supplement: S4 Fig — (A) The percentage change in body weight loss of WT and C3-/- mice over time. n = 7–12 mice per group. (B) Bacterial load in the striatum of WT, C3-/-, and C3aR-/- mice after 7-day infection by S. aureus. n = 4–8 mice per group. Data are represented as mean ± SEM. Two-way ANOVA with Tukey’s multiple-comparison test for (A). WT SA vs WT mock, *p < 0.05, ***p < 0.001, and ****p < 0.0001. C3-/- SA vs WT SA, #p < 0.05. One-way ANOVA with Dunnett’s multiple comparisons test for (B). **p < 0.01 and ****p < 0.0001. (TIF) [file ppat.1013126.s004.tif]
